# Exploiting Mass Spectrometry to Unlock the Mechanism of Nanoparticle-Induced Inflammasome Activation

**DOI:** 10.1021/acsnano.3c05600

**Published:** 2023-08-29

**Authors:** Govind Gupta, Jasreen Kaur, Kunal Bhattacharya, Benedict J. Chambers, Arianna Gazzi, Giulia Furesi, Martina Rauner, Claudia Fuoco, Marco Orecchioni, Lucia Gemma Delogu, Lars Haag, Jan Eric Stehr, Aurélien Thomen, Romain Bordes, Per Malmberg, Gulaim A. Seisenbaeva, Vadim G. Kessler, Michael Persson, Bengt Fadeel

**Affiliations:** † Institute of Environmental Medicine, Karolinska Institutet, 171 77 Stockholm, Sweden; ‡ Department of Medicine Huddinge, Karolinska Institutet, 141 52 Huddinge, Sweden; § Department of Biomedical Sciences, University of Padua, Padua 35121, Italy; ∥ Department of Medicine III and Center for Healthy Aging, TU Dresden, 01307 Dresden, Germany; ⊥ Department of Biology, University of Rome Tor Vergata, Rome 00173, Italy; # Division of Inflammation Biology, La Jolla Institute for Immunology, La Jolla, California 92037, United States; ∇ Department of Laboratory Medicine, Karolinska Institutet, 141 52 Huddinge, Sweden; ○ Department of Physics, Chemistry and Biology, Linköping University, 581 83 Linköping, Sweden; ◆ Department of Chemistry and Molecular Biology, University of Gothenburg, 412 96 Göteborg, Sweden; ¶ Department of Chemistry and Chemical Engineering, Chalmers University of Technology, 412 96 Göteborg, Sweden; ⧨ Department of Molecular Sciences, Swedish University of Agricultural Sciences, 750 07 Uppsala, Sweden

**Keywords:** cell death, inflammasome, mass spectrometry, monocyte, silica nanoparticles

## Abstract

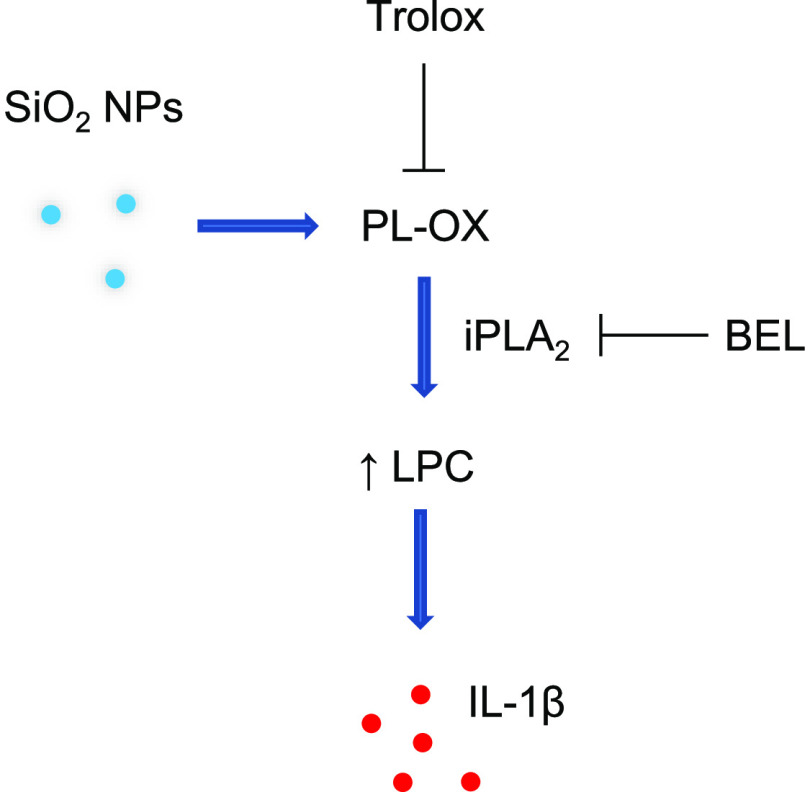

Nanoparticles (NPs) elicit sterile inflammation, but the underlying signaling pathways are poorly understood. Here, we report that human monocytes are particularly vulnerable to amorphous silica NPs, as evidenced by single-cell-based analysis of peripheral blood mononuclear cells using cytometry by time-of-flight (CyToF), while silane modification of the NPs mitigated their toxicity. Using human THP-1 cells as a model, we observed cellular internalization of silica NPs by nanoscale secondary ion mass spectrometry (nanoSIMS) and this was confirmed by transmission electron microscopy. Lipid droplet accumulation was also noted in the exposed cells. Furthermore, time-of-flight secondary ion mass spectrometry (ToF-SIMS) revealed specific changes in plasma membrane lipids, including phosphatidylcholine (PC) in silica NP-exposed cells, and subsequent studies suggested that lysophosphatidylcholine (LPC) acts as a cell autonomous signal for inflammasome activation in the absence of priming with a microbial ligand. Moreover, we found that silica NPs elicited NLRP3 inflammasome activation in monocytes, whereas cell death transpired through a non-apoptotic, lipid peroxidation-dependent mechanism. Together, these data further our understanding of the mechanism of sterile inflammation.

Inflammasomes are cytosolic multiprotein complexes in macrophages and other innate immune cells that are assembled following the detection of microbes or “danger” signals leading to the activation of caspase-1 with processing of pro-IL-1β.^1^ NLRP3 is an important and widely studied sensor of structurally diverse danger signals including nanoparticles (NPs).^2^ Seminal studies published 15 years ago revealed that crystalline silica, aluminum salts, and asbestos triggered the NLRP3 inflammasome, leading to the processing of pro-IL-1β and secretion of pro-inflammatory IL-1β.^3−5^ Subsequent work showed that amorphous silica NPs are also capable of triggering inflammasome activation.^6,7^ However, whether silica NP-induced cell death and inflammasome-dependent cytokine release are coordinately or separately regulated is unknown. For comparison, a recent study has shown that crystalline silica elicits pyroptosis, a caspase-mediated, gasdermin D/E-dependent cell death that is characterized by the release of IL-1β.^8^

Silica NPs produced through the high-temperature pyrolysis route (fumed silica) versus the low-temperature colloidal route were found to display differences in cytotoxic and pro-inflammatory potential.^9^ However, the molecular pathways of cytotoxicity (cell death) triggered by amorphous silica remain poorly understood.^10^ We reported in a previous study that small amorphous silica NPs caused cell death with glutathione (GSH) depletion and lipid peroxidation, which was reversed by overexpression of microsomal glutathione transferase 1 (MGST1), an antioxidant enzyme that has been implicated in the protection against lipid peroxidation.^11^ Here, we evaluated a set of amorphous silica NPs with varying surface properties with respect to cell death and inflammasome activation (Scheme S1). We could show that uncoated silica NPs triggered cell death in primary human monocytes; furthermore, we noted similarities with ferroptosis, a recently described form of non-apoptotic, lipid peroxidation-dependent cell death.^12^ Moreover, small, uncoated silica NPs triggered NLRP3 inflammasome activation in monocytes in the absence of priming with lipopolysaccharide (LPS), and this was shown to be regulated by lysophosphatidylcholine (LPC) downstream of plasma membrane remodeling. Previous studies have shown that silica NP-triggered inflammasome activation in macrophages is receptor-dependent,^13,14^ but our findings suggest that the signaling cascade culminating in inflammasome activation in monocytes is phagocytosis-independent. Hence, pro-inflammatory responses and ferroptosis-like cell death in response to amorphous silica NPs are decoupled, whereas the apical events, i.e., lipid peroxidation and subsequent remodeling of oxidatively damaged plasma membrane lipids, are evidently shared. These results increase our understanding of the cellular events underlying sterile inflammation.^15^

## Results and Discussion

### Characterization of Uncoated and Surface-Modified Silica NPs

Silica NPs were procured from a commercial source (Supporting Information Table S1). The NPs were uniform in size, as shown by TEM and SEM, and displayed particle diameters ranging from 12 to 100 nm (Figures S1 and S2); the smaller NPs displayed varying surface properties, i.e., pristine (uncoated) NPs versus Al-doped and silane-modified NPs. AFM confirmed the morphology of the silica NPs (Figure S3). The silane-modified NPs were produced by reacting alkyltrialkoxysilanes with the silanol groups on the silica surface. These NPs are thus sterically stabilized with glycerol–propyl moieties. Thermogravimetric analysis (TGA; Figure S1) and Fourier transform infrared spectroscopy (FTIR; data not shown) confirmed the successful silane modification of the NPs. Elemental analysis showed that the silica NPs were free of any impurities, apart from sodium, as the uncoated NPs were sodium-stabilized to improve the stability of the colloidal suspensions (Figures S1 and S2). Al (1.5%) was detected in the Al-doped NPs, in line with the surface modification of these particles. Dynamic light scattering (DLS) revealed that the hydrodynamic diameters of the NPs in culture medium supplemented with 10% fetal bovine serum (FBS) were comparable with the primary particle sizes (Table S1). The NPs displayed a negative surface charge, and the ζ potential values were decreased in the cell culture medium (Table S1). It is well-known that amorphous silica has the potential to generate reactive oxygen species.^10^ To uncover possible differences between the uncoated and silane-modified NPs, we performed electron paramagnetic resonance (EPR) spectroscopy. DMPO (5,5-dimethylpyrroline *N*-oxide) was used as a spin-trapping agent to detect transient free radicals. We observed characteristic DMPO-OOH^•^ adduct formation in the presence of H_2_O_2_, which further decayed to form DMPO-^•^OH, for both uncoated and silane-modified NPs (Figure S4), and similar results were obtained for the Al-doped silica NPs (data not shown), while no EPR signals were recorded for any of the silica NPs in the absence of H_2_O_2_.

### Single-cell Profiling of Immune Cell Impacts of Silica NPs

**Figure 1:**
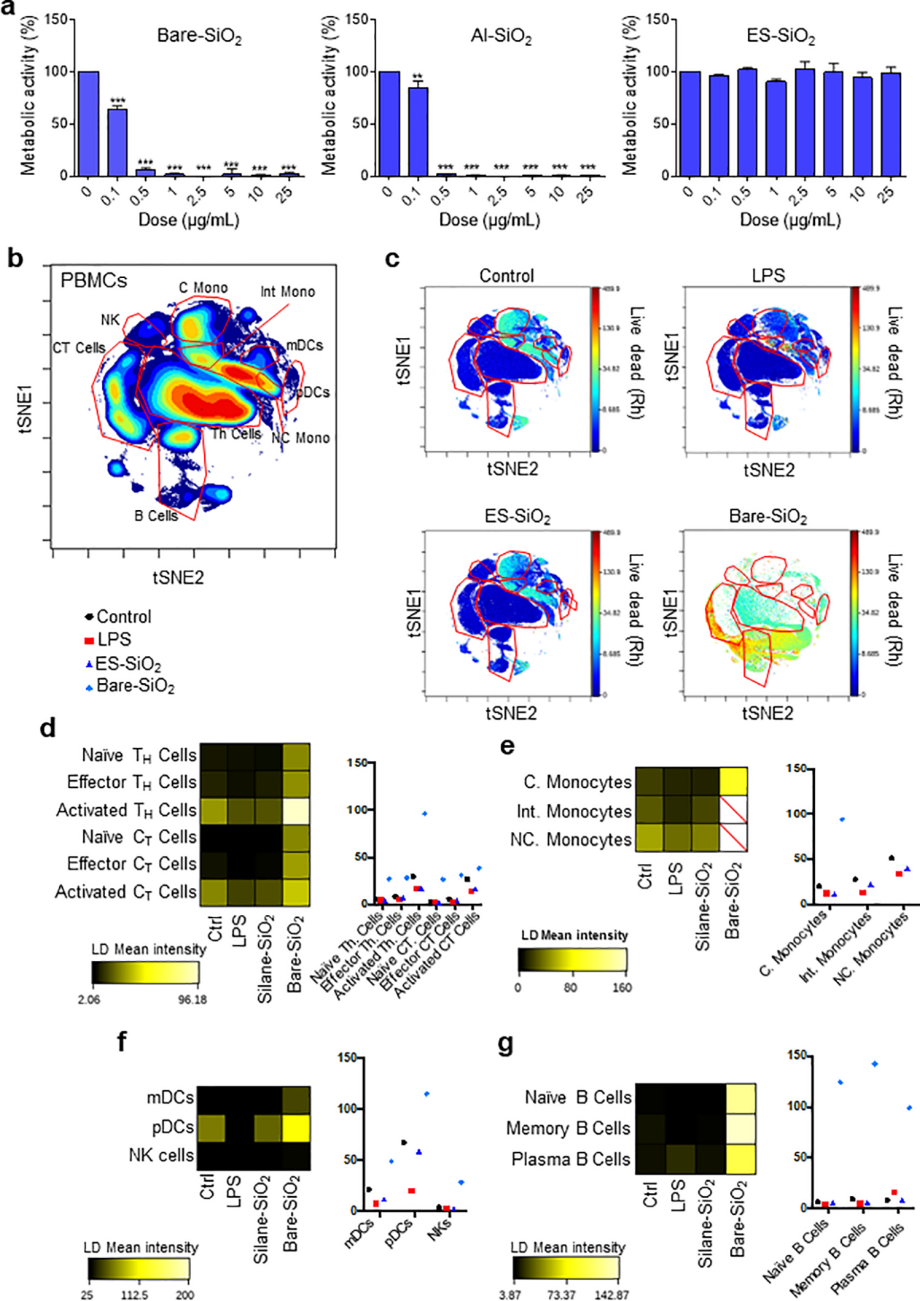
Single-cell mass cytometry of silica NP-exposed PBMCs. (a) Cell viability analysis of primary human monocytes after 24 h exposure to uncoated/bare, Al-doped, and silane-modified silica NPs. Data shown are mean values ± SD of experiments performed using cells from three independent donors. **p < 0.01; ***p < 0.001. (b) viSNE analysis depicting the single-cell characterization of PBMCs. For gating strategies for immune cell subpopulations, refer to Figure S5. (c) Single-cell analysis of cell viability by CyTOF. PBMCs were treated with bare or ethoxysilane (ES)-modified silica NPs at 0.1 μg/mL for 24 h. The viSNE plots show the different immune cell subpopulations for treated or untreated samples. LPS was used as a positive control. (d–g) Heat maps and histograms of rhodium mean marker expression ratios for gated T-cell subpopulations (d), monocyte subpopulations (e), DC and NK cell populations (f), and B cell subpopulations (g).

Single-cell mass cytometry or cytometry by time-of-flight (CyToF) is a powerful technology that enables the analysis of immune cell populations at the single-cell level, and we and others recently applied CyToF to study other nanomaterials.^16,17^ Here, we exploited the method to assess the impact of silica NPs on peripheral blood mononuclear cells (PBMCs). First, we performed a dose–response study using primary human CD14+ monocytes. The cells were exposed to uncoated versus surface-modified silica NPs for 24 h, and cell viability (metabolic capacity) was determined by using the Alamar blue assay (Figure 1a). Uncoated and Al-doped NPs triggered significant cytotoxicity, while silane-modified NPs were found to be completely non-cytotoxic at the doses tested (up to 25 μg/mL). Based on these results, we selected 0.1 μg/mL for the CyToF analysis. To this end, PBMCs were exposed to uncoated and silane-modified silica NPs for 24 h. Bacterial LPS was included as a positive control for immune cell activation. The different immune cell populations were identified based on the expression profiles of the various cluster of differentiation (CD) markers (Figure S5). Cell-ID Intercalator-103Rh staining was performed to assess the cell viability. To visualize the high-dimensional expression profiles, we applied viSNE,^18^ an implementation of the t-stochastic neighbor embedding (tSNE) algorithm (Figure 1b). The viSNE maps in Figure 1c clearly showed that the most evident loss of events occurred in the monocyte compartment with high-LD mean intensity values for classical monocytes and a complete loss of events for intermediate and nonclassical monocytes following exposure to the uncoated silica NPs. In addition, activated T helper cells, plasmacytoid dendritic cells (pDCs), and several B-cell subpopulations displayed high-LD mean intensity values in response to uncoated silica NPs (Figure 1d–g). It is worth noting that so-called ultrasmall silica NPs were shown by others to trigger dose-dependent CD4 and CD8 T-cell activation.^19^ Natural killer (NK) cells, on the other hand, were not affected at 0.1 μg/mL (Figure 1f). Meanwhile, the silane-modified NPs were non-cytotoxic across all of the cell populations. We also investigated single-cell profiles for selected cytokines in response to silica NPs, and found that the uncoated silica NPs, but not the silane-modified NPs, triggered IL-2 production in various B-cell populations and DCs. In addition, uncoated silica NPs upregulated IL-6, a pro-inflammatory cytokine, in monocytes (Figure S6). To further address whether NK cells are susceptible or not to silica NPs, we isolated CD56+ NK cells and exposed the cells to uncoated versus surface-modified silica NPs for 24 h (Figure S7a–c). No cell death was recorded at 0.1 μg/mL. However, uncoated and Al-doped NPs both triggered cell death (loss of metabolic capacity) at doses higher than 0.1 μg/mL, while the silane-modified silica NPs were completely non-cytotoxic. We also evaluated monocytes versus NK cells with respect to IL-1β secretion and found that uncoated silica NPs triggered IL-1β secretion only in monocytes (Figure S7d). Hence, while uncoated silica NPs were strongly cytotoxic toward monocytes, silane modification mitigated this effect. Other investigators have reported that aluminum and titanium doping of fumed silica NPs could reduce surface silanol density, resulting in a reduction in hydroxyl radical generation and cytotoxicity in THP-1 cells.^20^ Furthermore, titanium doping ameliorated pulmonary inflammation and fibrosis in silica NP-exposed mice.^21^ Taken together, surface properties are shown to play a key role in the toxicity of silica NPs.

### Label-Free Detection of Cellular Interactions of Silica NPs

**Figure 2:**
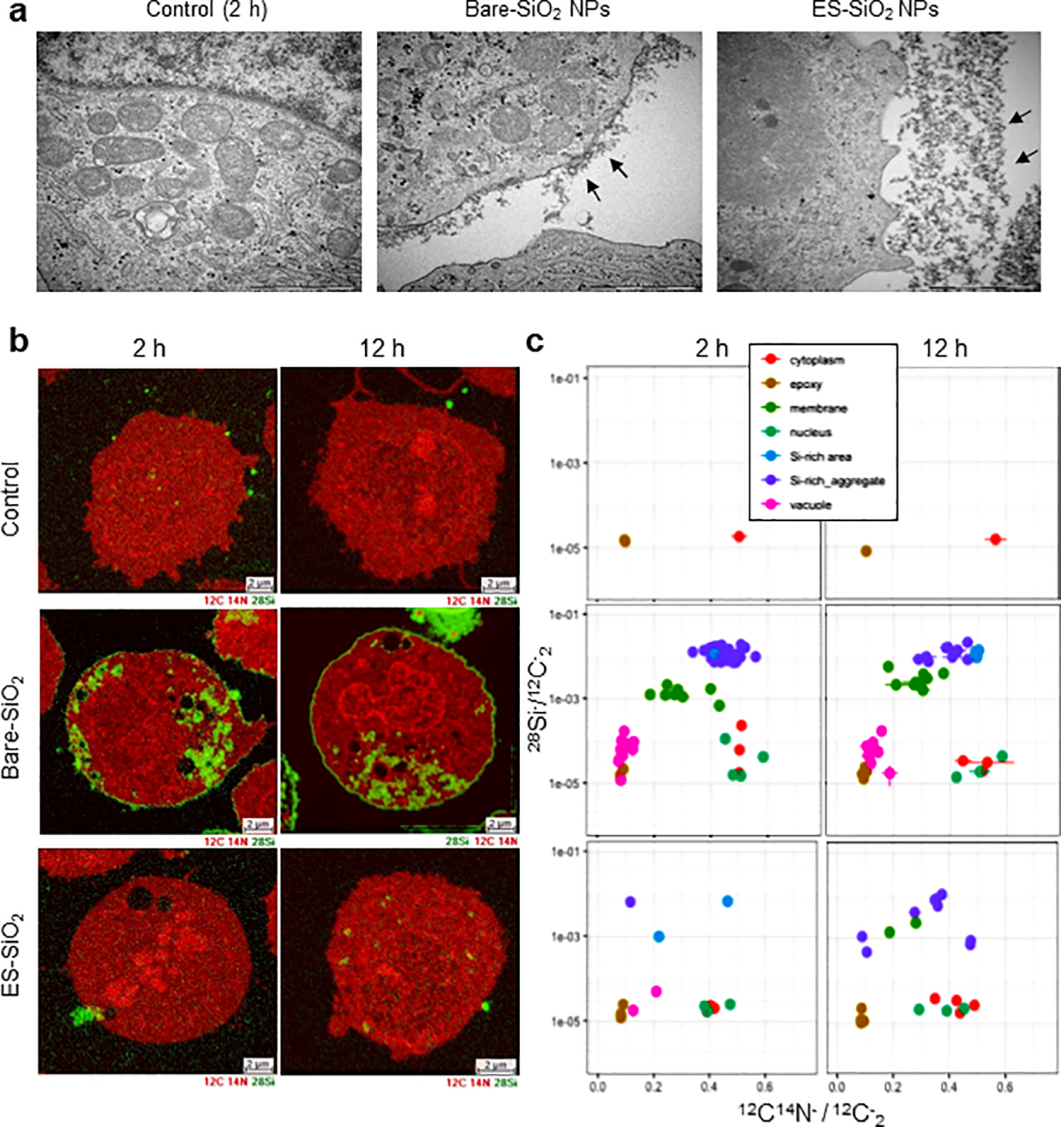
Cellular interaction and uptake of small silica NPs. (a) TEM images showing cell surface interactions of bare versus silane-modified silica NPs at 2 h. Arrows point to NPs on the cell membrane or outside the cells. For results on other silica NPs of different primary particle sizes, refer to Figure S8. (b) Label-free nanoSIMS analysis of THP-1 cells exposed to bare and silane-modified silica NPs for 2 and 12 h. (c) Quantification of relative abundance of silica NP distribution in cells at 2 and 12 h, based on nanoSIMS.

To study the impact of silica NPs on monocytes in more detail, we decided to use the human THP-1 cell line, a commonly applied model of monocytes.^22^ First, we performed TEM analysis of cells for 2 h to elucidate cellular interactions of the silica NPs. The results revealed interactions of the uncoated silica NPs with the cell surface, while the silane-modified NPs did not seem to interact with the cells (Figure 2a). However, cellular internalization was difficult to prove due to the small size of these particles (i.e., similar in appearance to ribosomes). Using a set of unmodified silica NPs of increasing sizes, we could confirm uptake by TEM analysis of THP-1 cells (Figure S8). The NPs were found to reside in membrane-enclosed vesicles, suggestive of endocytosis (at least for the larger sized particles). To unequivocally demonstrate cellular internalization of the small silica NPs, we employed nanoSIMS, a technique based on the simultanous collection of multiple secondary ions from sample surfaces to create elemental maps at high lateral resolution (50 nm) and with high sensitivity (ppm in element imaging).^23^ Shown in Figure 2b are nanoSIMS composite images of ^12^C^14^N^–^ and ^28^Si^–^ ion maps from THP-1 cells exposed to 2.5 μg/mL uncoated and silane-modified silica NPs for 2 and 12 h. We thus noted a strong ^28^Si^–^ signal in cells exposed to uncoated silica NPs compared with the control. Notably, no signal or a very weak ^28^Si^–^ signal was detected in cells exposed to silane-modified NPs. Furthermore, relative quantification of the ^28^Si^–^ signal indicated that aggregates of silica NPs were abundant in the cytoplasm as well as in/on the plasma membrane of THP-1 cells (Figure 2c).

Macrophage uptake of inorganic NPs was previously reported to occur via scavenger receptors.^24,25^ To address whether silica NPs are internalized in a similar manner in our model, we prepared fluorescent silica NPs in which FITC was incorporated into the core of the particle, rendering the surface of the NPs identical to that of the unmodified silica NPs. Evidence for cellular uptake of the fluorescent NPs (50 μg/mL) was obtained using confocal microscopy (Figure S9a). To evaluate the role, if any, of the scavenger receptors for cellular uptake of the NPs, THP-1 cells were exposed for 6 h to silica NPs in the presence or absence of fucoidan, a scavenger receptor inhibitor.^26^ However, fucoidan did not reduce cell uptake (Figure S9b). Taken together, clear evidence for plasma membrane interaction/adherence was observed for uncoated silica NPs in our monocyte-like model. Cellular uptake, likely receptor-independent, was also observed by TEM and nanoSIMS.

### Silica NPs Promote Cytoplasmic Lipid Droplet Formation

**Figure 3:**
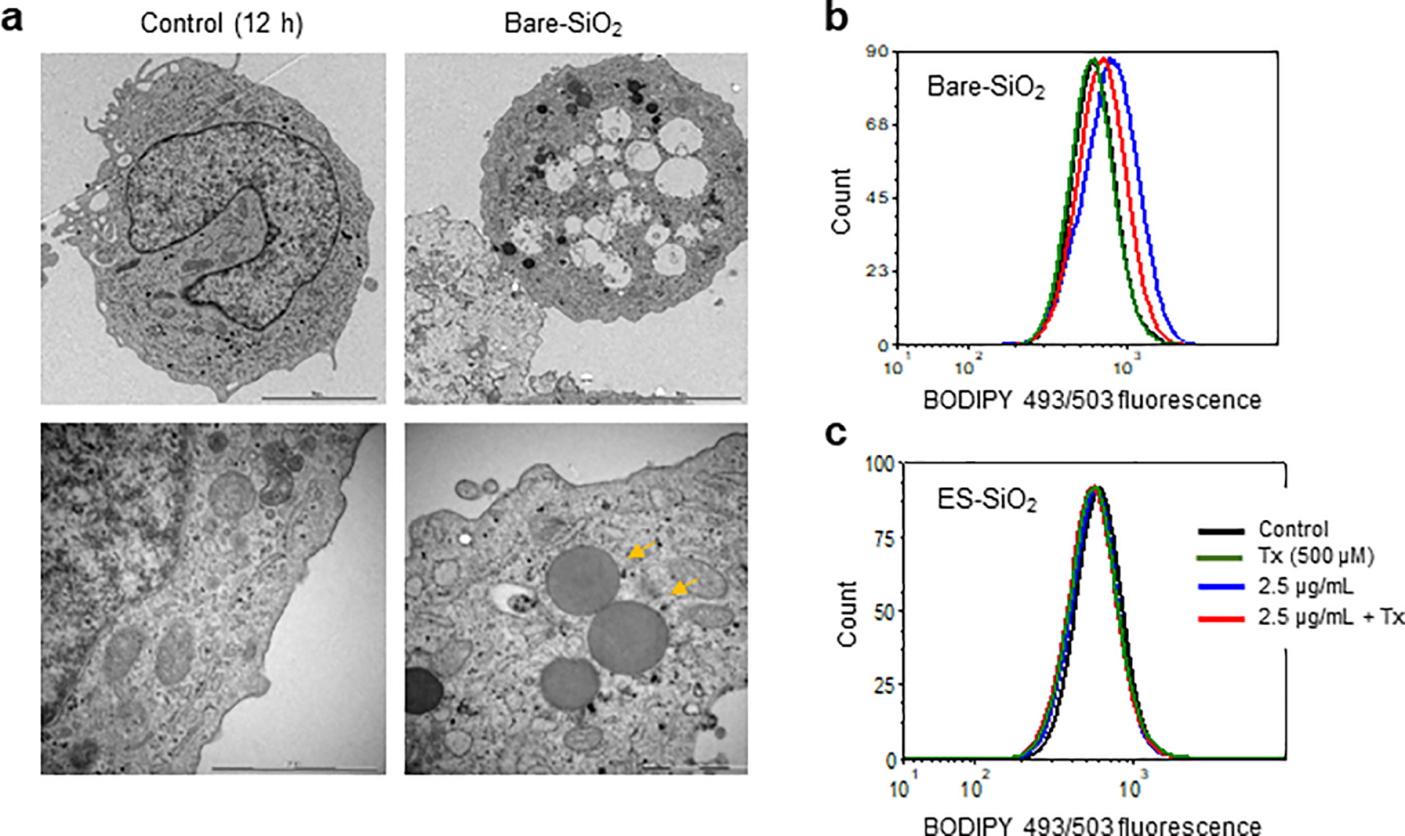
Silica NPs trigger cytoplasmic lipid droplet formation. (a) TEM images showing ultrastructural changes in THP-1 cells exposed for 12 h to bare silica NPs versus control cells. Cytoplasmic vacuolization (upper panel) and lipid droplet accumulation (arrows, lower panel) were noted. (b, c) Lipid droplet content quantified using flow cytometry after labeling the cells with BODIPY 493/503. The cells were exposed to silica NPs in the presence or absence of Trolox. For further results using Nile Red staining, see Figure S10.

To further evaluate the impact of silica NPs, and to complement the results obtained at 2 h, we examined THP-1 cells by TEM following exposure to silica NPs for 12 h. The uncoated silica NPs were found to elicit the formation of large cytoplasmic vacuoles (Figure 3a). Cytoplasmic vacuolization is a common response to offending microorganisms as well as inorganic nanoparticles.^27^ Moreover, lipid droplets featured prominently in cells exposed to silica NPs (Figure 3a). This was not observed in control cells. Using the lipophilic dye Nile Red, we confirmed the presence of lipid droplets in NP-exposed cells (Figure S10a). Lipid droplets are highly dynamic organelles with key roles in the regulation of lipid storage and lipid metabolism, and studies have shown that lipid droplets are also involved in the protection against cell death through the sequestration of polyunsaturated fatty acids (PUFAs).^28^ To provide further evidence of lipid droplets in silica-NP-exposed cells, cells were labeled with BODIPY 493/503, a lipid-droplet-specific fluorescent dye. The BODIPY 493/503 fluorescence increased upon exposure to uncoated silica NPs, and this was decreased in cells pre-incubated with Trolox, a water-soluble analog of vitamin E (Figure 3b). On the other hand, BODIPY 493/503 staining was not affected in cells exposed to the silane-modified silica NPs (Figure 3c). Pre-incubation with a DGAT (diacylglycerol acyltransferase) inhibitor prevented silica-NP-induced lipid droplet formation, thus confirming the specificity of the flow cytometry-based assay (Figure S10b). Taken together, it is probable that lipid droplets play an antioxidant role^29^ in silica-exposed cells.

### Silica-NP-Induced Cell Death Is Lipid Peroxidation-Dependent

We evaluated the toxicity of silica NPs toward THP-1 cells using the Alamar blue assay (metabolic capacity). The uncoated silica NPs were strongly cytotoxic. The Al-doped silica NPs displayed a similar profile, while no cytotoxicity was recorded for the silane-modified NPs, in line with the results obtained for primary monocytes. The larger uncoated NPs were found to be less cytotoxic (data not shown). Because our EPR analysis showed that the propensity for radical formation is similar for uncoated and silane-modified NPs, we favor the view that the lower density of free silanol groups on the silane-modified NPs is the reason why these NPs are less cytotoxic. Additionally, the latter NPs are stabilized with flexible glycerol–propyl tails, further decreasing the likelihood of interactions between the silanol groups and the cell membrane. To investigate the role of sodium in the sodium-stabilized NPs, we proceeded to evaluate the cytotoxicity of ion-exchanged silica NPs. Dose-dependent cytotoxicity was observed as being comparable to that seen for the original NPs (Figure S11a,b). To assess the putative role of soluble silica monomers, the NPs were “aged” for 7 days in Milli-Q water or in cell culture medium with or without 10% FBS. The presence of serum was previously shown to mitigate the cytotoxicity of certain silica NPs.^11,30^ However, the protein corona may preferentially affect NPs that otherwise tend to agglomerate, whereas colloidal silica was addressed in the present study. In fact, no differences in cytotoxicity were observed with or without FBS for aged or fresh NPs (Figure S11c–e). Thus, the cytotoxicity of the uncoated NPs cannot be explained by impurities in the samples and occurred independently of whether serum was present or not.

The mechanism of silica NP-induced cell death remains poorly understood.^31^ To study markers of cell death, we applied C11-BODIPY 581/591, a fluorescent probe for lipid peroxidation in cells. We observed a dose-dependent lipid peroxidation in cells exposed for 6 h to small, uncoated silica NPs (Figure S12a), which was alleviated by Trolox. Moreover, the exposure of cells to uncoated silica NPs drastically reduced cellular GSH levels, and this was also reversed by Trolox (Figure S12b). To probe the mechanism of cell death, we asked whether inhibitors of known cell death modalities would affect silica NP-triggered cell death (at 12 h). Notably, cell death induced by the uncoated silica NPs was unaffected by zVAD-fmk (Figure S12c), thus excluding caspase-dependent apoptosis. Necrostatin-1, a RIPK1 inhibitor that blocks necroptosis, and Ferrostatin-1, a radical scavenger that is commonly used to block ferroptosis, also failed to prevent silica-induced cell death (data not shown). Moreover, Ca074Me did not rescue THP-1 cells exposed to the uncoated silica NPs, suggesting that lysosomal cathepsins are not involved (Figure S12d). Ferroptosis is characterized by lipid peroxidation and its vulnerability to lipid antioxidants such as Trolox as well as to the iron-chelating agent, deferoxamine (DFO).^32^ However, DFO failed to block cell death triggered by silica NPs (2.5 μg/mL; Figure S12e). Hence, silica-NP-induced cell death can be prevented by the vitamin E analog Trolox and by overexpression of the GSH-dependent antioxidant enzyme MGST1, as we have reported previously for other small amorphous silica NPs,^11^ but does not seem to correspond to the known forms of regulated cell death. Nonetheless, it can be concluded that the uncoated silica NPs trigger a lipid-peroxidation-dependent cell death resembling ferroptosis, albeit without a requirement for labile (chelatable) iron.

### Silica NPs Trigger Specific Changes in Plasma Membrane Lipids

Having established that the uncoated silica NPs triggered lipid peroxidation-dependent cell death, and in view of the membrane damaging potential of silica NPs,^33,34^ we sought to investigate whether the uncoated silica NPs elicited specific changes in the plasma membrane. To this end, we utilized time-of-flight secondary ion mass spectrometry (ToF-SIMS), a surface analytical tool that is highly suited to the detection of changes in plasma membrane lipids.^35^ To this end, we compared unexposed cells and cells exposed for 2 and 6 h to uncoated or silane-modified silica NPs (2.5 μg/mL), in the presence or absence of Trolox. Previous work has shown that ToF-SIMS can be applied to detect and laterally resolve silica NPs in biological samples.^36^ Indeed, we observed the presence of uncoated silica NPs in the cell membrane on the basis of the increased intensity of silica at *m*/*z* 28.00 with respect to the control; this was unaffected by Trolox (Figure S13). However, we could not detect changes in the *m*/*z* 28.00 intensity with respect to the control following exposure to the silane-modified silica NPs, in line with our nanoSIMS results (see above). The CN^–^ signal (*m*/*z* 26.00) can be used as a pseudo-optical image of protein-rich structures, while the phosphate signal (PO_3_-, *m*/*z* 78.96) may serve as an indicator of overall lipid content.^37^ ToF-SIMS images showed a decrease in the intensity of PO_3_-signals upon exposure to uncoated NPs, while cells exposed to silane-modified NPs showed no decrease in the PO_3_-content (Figure S13). Principal component analysis (PCA) is the most common form of analysis of ToF-SIMS data. Here, the samples are mass spectra and the variables are *m*/*z* channels within the spectra. We performed PCA on the normalized spectra obtained in positive and negative ion mode from each experimental group and observed a clear separation using the first component (t[1]) versus the second component (t[2]). Hence, the groups were well-separated and the changes in lipid content were sufficient to chemically differentiate controls and samples exposed to the uncoated silica NPs (Figure S14).

**Figure 4:**
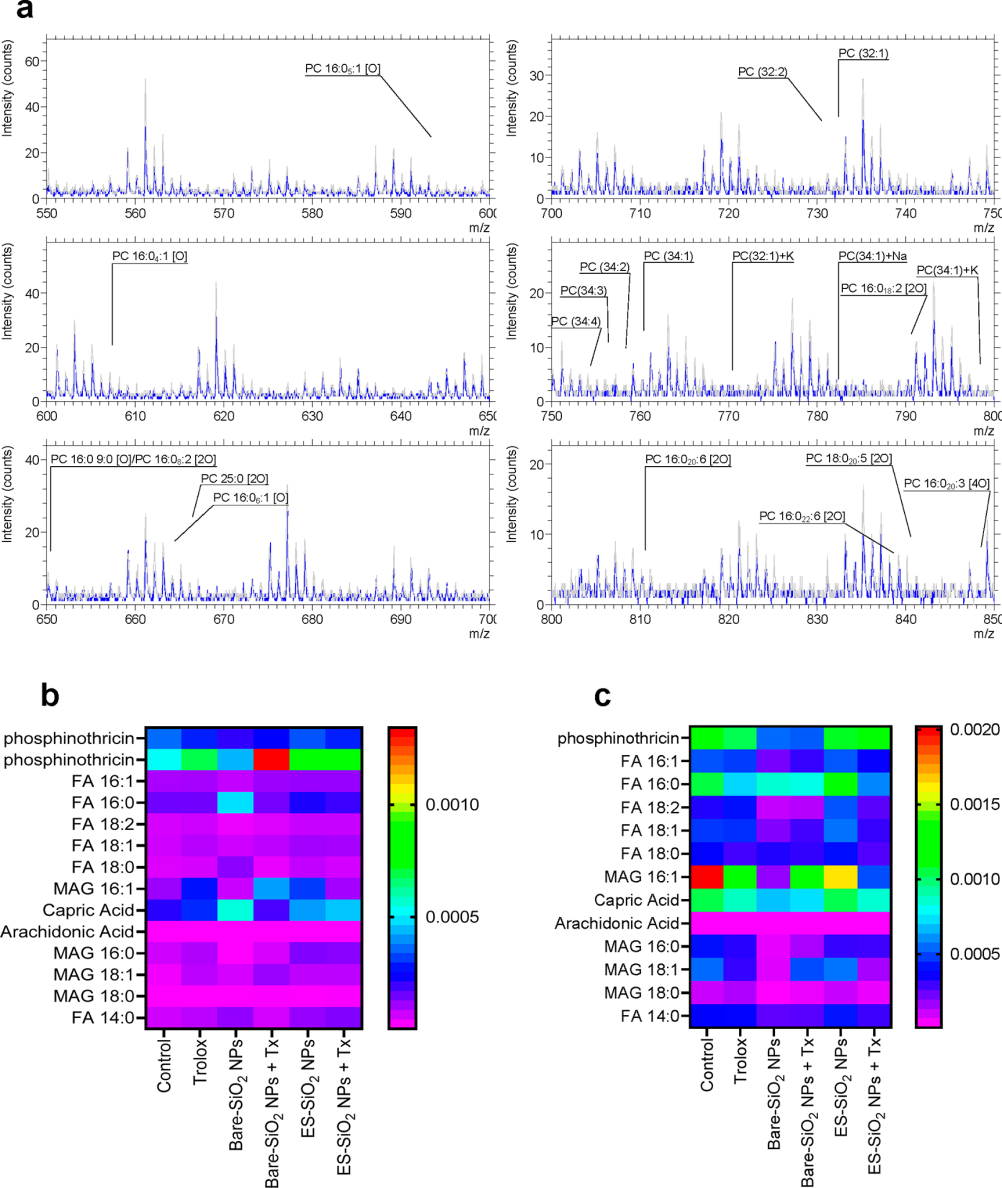
Silica NP-induced membrane lipid changes analyzed by ToF-SIMS. (a) ToF-SIMS mass spectra recorded in positive ion (*m*/*z* 550 to *m*/*z* 850) indicating that phosphatidylcholine (PC) and its fragments were decreased after exposure for 6 h to small (12 nm) bare SiO_2_ NPs (blue) with respect to control (untreated cells) (gray). (b, c) Heat maps showing normalized intensity values of *sn*-1 and *sn*-2 acyl chain fatty acids detected in negative ion mode at 2 and 6 h of exposure, respectively. THP-1 cells were exposed to bare and ethoxysilane (ES)-modified silica NPs in the presence and absence of Trolox, as indicated. Refer to Figure 5 for quantification of the data obtained at 6 h.

**Figure 5:**
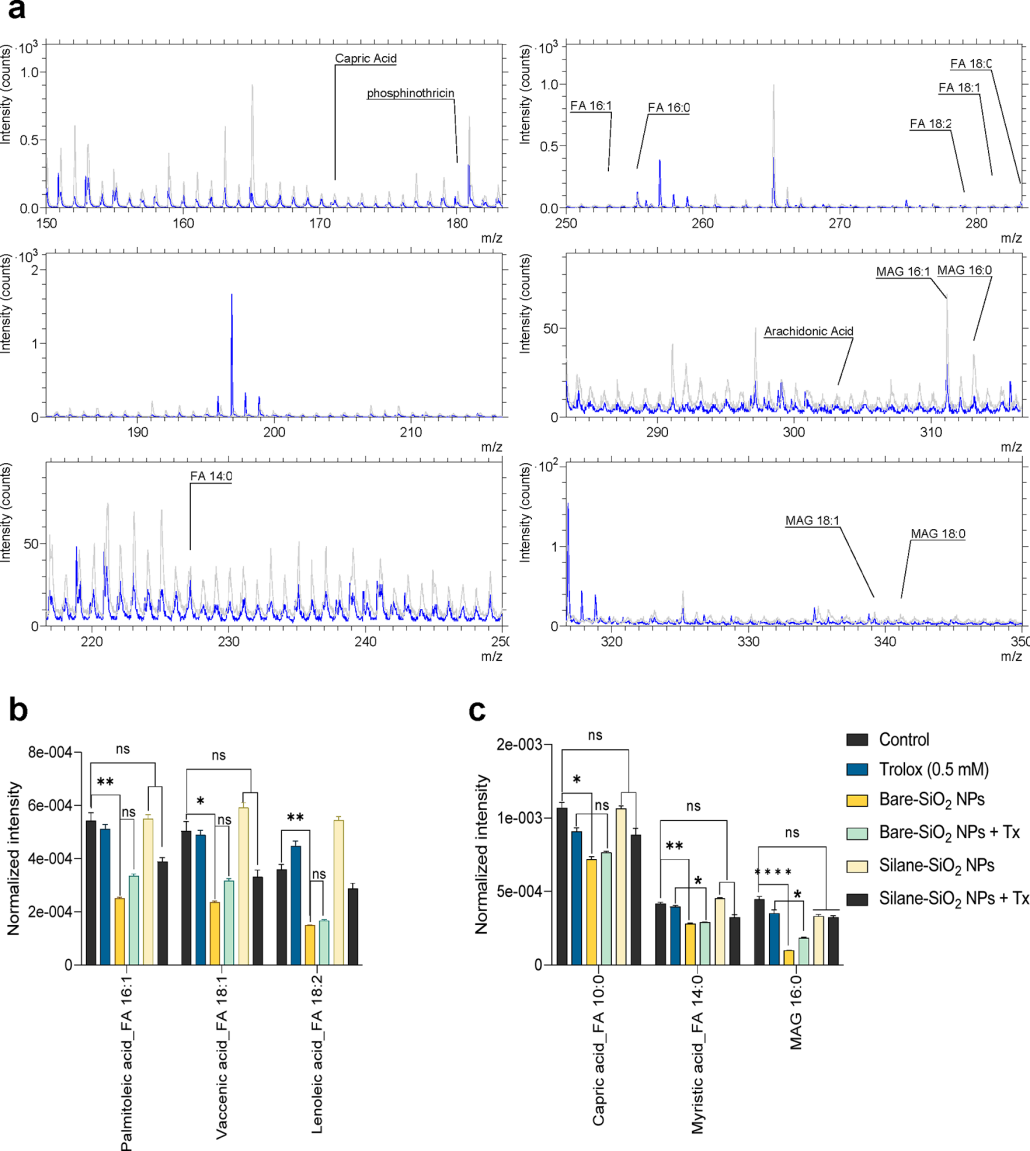
ToF-SIMS reveals changes in fatty acids in the plasma membrane of silica NP-exposed cells. (a) ToF-SIMS mass spectra recorded in negative ion mode (*m*/*z* 150 to *m*/*z* 350) indicating a change in various fatty acids at 6 h after exposure to small, bare SiO_2_ NPs (blue) with respect to control (gray). (b, c) Most significantly altered unsaturated and saturated fatty acids, respectively, following exposure of THP-1 cells to bare versus silane-modified (ES) silica NPs for 6 h, in the presence or absence of Trolox. The results in panels b and c are shown as mean values ± SD (*n* = 4). *p < 0.05; **p < 0.01; ****p < 0.0001.

Next, changes in major phospholipids and fatty acids in the plasma membrane were compared among the different groups. The most significant (*p* < 0.05) change following the exposure of cells to the uncoated silica NPs for 6 h was a decrease in phospholipids, specifically, phosphatidylcholine (PC, *m*/*z* 184 and 224), phosphatidylethanolamine (PE, *m*/*z* 140 and 180), and phosphatidylinositol (PI, *m*/*z* 153 and 241). PC was the most abundant phospholipid detectable by ToF-SIMS (Figure 4a) and its intensity decreased uniformly for all of the fragments of PC detected between *m*/*z* 550 and 850 in uncoated silica NP exposed cells, while no changes were observed in cells exposed to the silane-modified NPs. Trolox protected from loss of PC after exposure to the uncoated silica NPs. Furthermore, we compared the changes in fatty acids after exposure to uncoated versus silane-modified silica NPs (Figure 5a). Hence, the intensity of saturated fatty acids was increased, while unsaturated fatty acids were decreased upon exposure for 2 h to uncoated silica NPs while the silane-modified silica NPs did not elicit any changes in fatty acid composition. We also noted a decrease in saturated and unsaturated monoacylglycerols (MAGs) (16:0 and 16:1) after exposure to the uncoated silica NPs (Figure 4b). Among the investigated saturated fatty acids, palmitic acid (FA 16:0, *m*/*z* 255), capric acid (*m*/*z* 171), and myristic acid (FA 14:0, *m*/*z* 227) were the ones most affected. With regard to the unsaturated fatty acids, palmitoleic acid (FA 16:1, *m*/*z* 253) and linoleic acid (FA 18:2, *m*/*z* 274) were the most affected. Furthermore, at 6 h, a marked decrease in both saturated and unsaturated fatty acids was observed upon exposure to uncoated silica NPs with respect to control cells (Figure 4c). Again, palmitoleic acid and linoleic acid were the most highly affected unsaturated fatty acids, and capric acid and myristic acid were the most affected saturated fatty acids at 6 h (Figure 5b,c). MAGs were more significantly decreased at 6 h compared to 2 h. To sum up, silica is thought to have a high propensity to interact with lipids, specifically, with the phosphate groups of phospholipids such as PC,^38^ and the present study has provided direct evidence for such effects in human cells exposed to uncoated (or “bare”) silica NPs.

### Silica NPs Trigger IL-1β Production without Cell Priming

**Figure 6:**
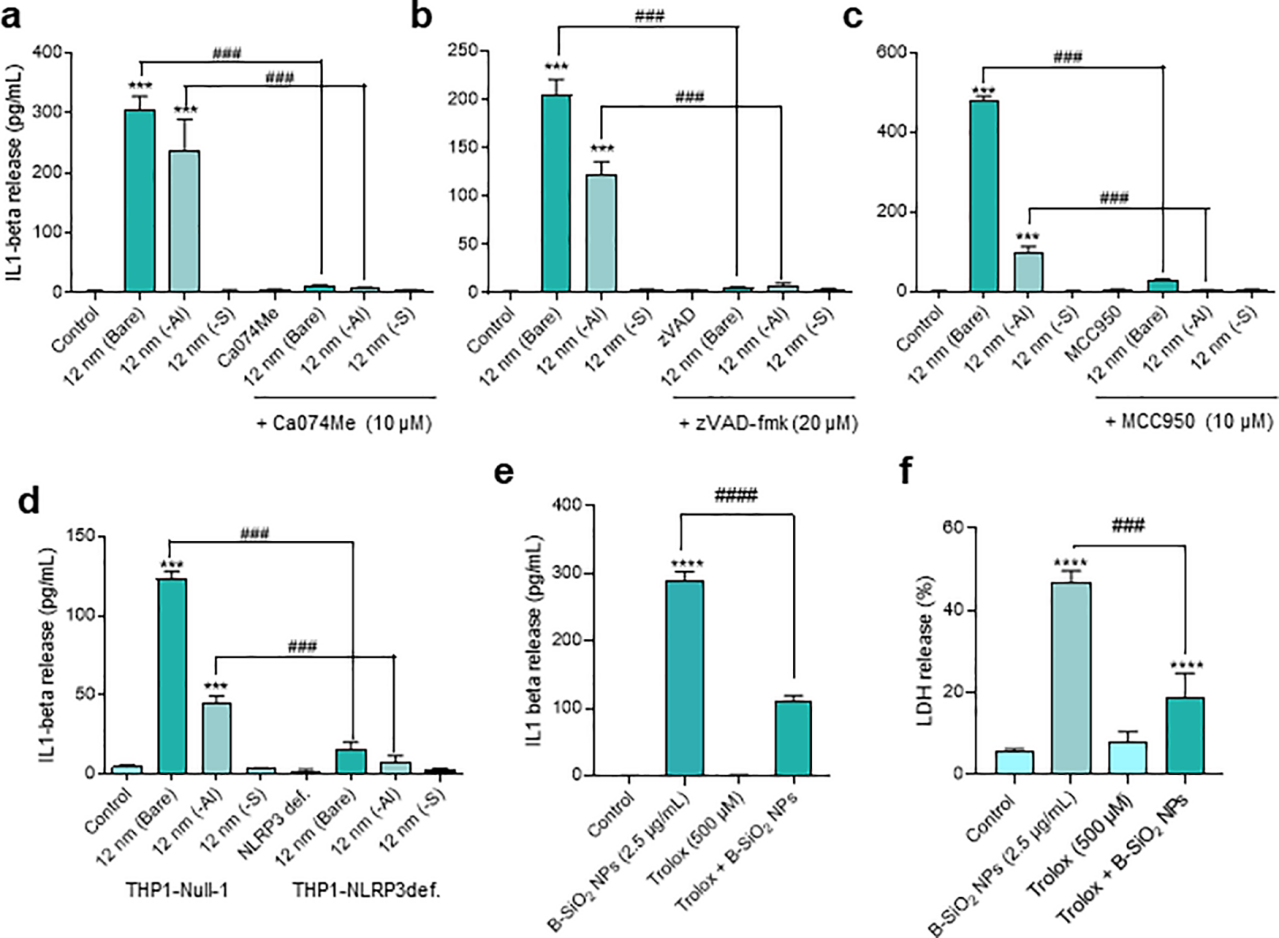
Silica NPs trigger NLRP3-dependent IL-1β secretion. IL-1β release triggered by bare and Al-doped silica NPs is blocked by the cathepsin B inhibitor, CA074Me (a), the pan-caspase inhibitor, zVAD-fmk (b), and MCC950, a selective inhibitor of the NLRP3 inflammasome (c). For results on other silica NPs of varying sizes as well as the benchmark NPs, refer to Figure S16. Note that the cells were nonprimed. For results using LPS-primed cells, refer to Figure S17. The role of the NLRP3 inflammasome (in the absence of LPS priming) was further confirmed by using wild-type (Null-1) and NLRP3-deficient THP-1 cells (d). For results using caspase-1-deficient cells, refer to Figure S18. (e) Lipid antioxidant Trolox significantly reduced IL-1β release triggered by bare silica NPs. (f) Cell death was also significantly reduced by Trolox, while cell death unaffected by cathepsin B or caspase inhibitors (see Figure S12). Data are shown as mean values ± SD (*n* = 3). ***p < 0.001; ###*p* < 0.001; ****p < 0.0001; #*p* < 0.05; ####*p* < 0.0001.

The NLRP3 inflammasome responds not only to microbial stimuli but also to a wide range of metabolic stressors such as uric acid or cholesterol as well as to exogenous substances (e.g., asbestos, alum, quartz).^1^ However, most in vitro studies of NLRP3 inflammasome activation have employed a protocol of LPS priming to trigger inflammasome activation.^39,40^ In fact, even in studies where bacterial toxins and particulate matter were compared with respect to inflammasome activation, cells were first primed with LPS (a component of the bacterial cell wall).^41^ Other investigators have invoked purinergic signaling in particulate matter-induced inflammasome activation^42^ and in cells exposed to silica NPs.^43^ However, exceptionally high doses of NPs (250 μg/mL) were applied in the latter study (100 times higher than in the present study), and the results must be interpreted with caution. To investigate whether silica NPs triggered pro-inflammatory responses, we determined IL-1β secretion in THP-1 cells following exposure to NPs (2.5 μg/mL) in the absence of priming. The uncoated and Al-doped silica NPs triggered IL-1β secretion at 6 h, whereas the silane-modified NPs failed to trigger IL-1β secretion under these conditions (Figure 6a). IL-1β production was not affected by cytochalasin D, an inhibitor of actin polymerization,^44^ suggesting that active cellular uptake of the NPs was not required (data not shown). Phagocytosis-independent release of IL-1β has been reported previously for other NPs.^7^ However, these findings do not preclude other means of cellular uptake. Indeed, nanoSIMS revealed the presence of silica within the cells already after 2 h (see above). Moreover, no TNF-α release was observed in THP-1 cells or in primary human monocytes exposed for 6 h to uncoated, aluminized, or silane-modified NPs (Figure S15a,b), thus demonstrating that the observed effects were specific for IL-1β production.

We observed a size-dependent IL-1β secretion (in the absence of priming) insofar as the small, uncoated NPs triggered IL-1β production, while this was not the case for the larger (100 nm) silica NPs (Figure S16a). We also tested two “benchmark” silica NPs from the JRC (NM200 and NM203) (Table S1). We have previously shown that these NPs are non-cytotoxic toward nondifferentiated THP-1 cells.^45^ We found that only NM200 (50 μg/mL) triggered IL-1β secretion and only in the presence of LPS priming (Figure S16b,c). Furthermore, aging of the particle dispersions for 18 months was performed to evaluate the potential role of silica monomers, but this had a minor impact on the inflammogenic properties of the particles. It is important to note that nondifferentiated (monocyte-like) THP-1 cells were used for these experiments (along with primary human monocytes; see above). Previous work has shown that monocytes and macrophages differ in terms of inflammasome engagement (in response to LPS).^46^ Thus, in order to address whether monocyte-like and macrophage-like cells respond differently to silica NPs and whether LPS priming plays a role, we performed experiments in which THP-1 cells were maintained as nondifferentiated cells or were differentiated into macrophage-like cells by using the phorbol ester PMA.^47^ We found that the uncoated and Al-doped NPs triggered IL-1β production in monocyte-like cells, and this response was enhanced following LPS priming for 2 h prior to the addition of the NPs (Figure S17a). However, the silane-modified NPs failed to elicit a response (with or without LPS priming). Using macrophage-like cells, a similar trend was observed but the levels of IL-1β production were higher (Figure S17b). The silane-modified NPs did not trigger a response compared to LPS priming alone, while the response to the uncoated and Al-doped NPs was significantly enhanced in the presence of LPS priming, in line with available literature on other nanomaterials.^48^

### Silica NPs Trigger NLRP3 Inflammasome Activation without Cell Priming

Next, we asked whether silica NP-triggered IL-1β production was due to NLRP3 inflammasome activation. Indeed, we found that the small uncoated silica NPs triggered IL-1β secretion in a cathepsin B-dependent (Figure 6a) as well as caspase-dependent manner (Figure 6b). Furthermore, MCC950, a selective NLRP3 inhibitor, also blocked IL-1β secretion elicited by uncoated silica NPs (Figure 6c). Additionally, by using wild-type versus NLRP3-deficient nondifferentiated THP-1 cells, we confirmed that silica NP-induced IL-1β secretion was, indeed, NLRP3-dependent (Figure 6d). Similarly, the silica-NP-triggered response was nullified in caspase-1-deficient THP-1 cells (Figure S18a). In contrast, pre-incubation of cells with A438079, a competitive P2X_7_ antagonist, failed to block IL-1β secretion, suggesting that purinergic signaling is not involved in the current model (data not shown). Thus, small, uncoated silica NPs triggered canonical inflammasome-dependent IL-1β production in the monocyte-like THP-1 cell line in the absence of priming with a microbial ligand. For comparison, nigericin, a prototypic pyroptosis inducer,^41^ prompted IL-1β production in LPS-primed cells but failed to do so in nonprimed cells (Figure S18b,c). We then asked whether lipid peroxidation plays a role in the silica NP-induced IL-1β production. Indeed, Trolox significantly dampened silica NP-induced IL-1β release (Figure 6e). Cell death was also attenuated by Trolox (Figure 6f). Hence, although silica NP-induced cell death and inflammasome activation differ in terms of the involvement of cathepsins and caspases, both are regulated by lipid peroxidation.

Overall, the propensity of silica NPs to interact with phospholipids,^49,50^ and the capacity for hydroxyl radical formation,^9^ may explain the membrane damage evidenced in silica NP-exposed cells. However, it is important to consider whether cellular ROS also play a role, as several previous studies have shown that silica NPs provoke ROS production.^51,52^ One conundrum is that such effects are thought to require the active uptake of the NPs through endocytosis. However, it is possible that a proportion of NPs could passively cross the plasma membrane.^53^ Notwithstanding, we have shown that silica NPs of various sizes are internalized by cells, and we also found that uncoated silica NPs but not their silane-modified counterparts triggered ROS production as early as 1 h after exposure (Figure S19a,b), while MitoTEMPO, a selective scavenger of mitochondrial ROS, attenuated silica-NP-triggered cell death (albeit not significantly) (Figure S20a). Therefore, it is conceivable that mitochondrial ROS provide the match that ignites the silica-induced membrane damage seen here for the uncoated but not the silane-modified NPs.

### Silica NP-Induced IL-1β Production Is Modulated by LPC

**Figure 7:**
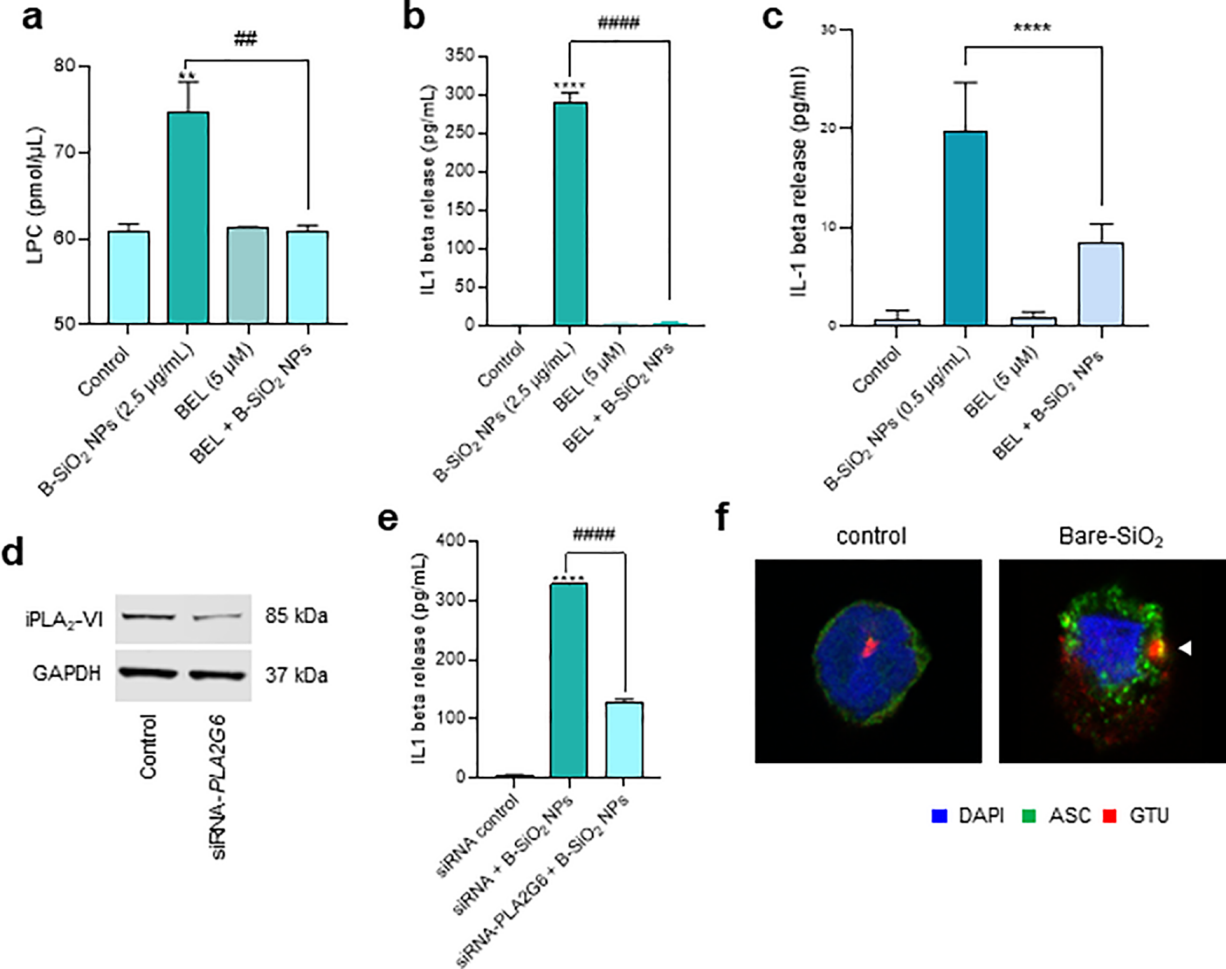
Pro-inflammatory responses of silica NPs are iPLA_2_-dependent. (a) Bare silica NPs triggered an elevation in cellular LPC content in THP-1 cells, which was prevented by BEL, a selective iPLA_2_-VIA inhibitor. Cells were exposed for 6 h to bare silica NPs (2.5 μg/mL) with or without BEL (5 μM). (b) Silica NP-triggered IL-1β release in nondifferentiated (monocyte-like) THP-1 cells was effectively blocked by BEL. (c) Silica NP-triggered IL-1β release in primary human CD14+ monocytes was also reduced by BEL. (d) Western blot to confirm *PLA2G6* silencing in THP-1 cells using specific siRNAs. GAPDH was used as a loading control. (e) Silencing of *PLA2G6* significantly reduced IL-1β production as determined by ELISA. (f) Silica NPs triggered inflammasome assembly at the MTOC, as evidenced by the colocalization of ASC and the centrosomal marker, γ-tubulin (GTU). Samples obtained after 6 h of exposure were visualized by confocal microscopy. For additional results on the impact of BEL, refer to Figure S20. Data shown as mean values ± SD (*n* = 3). **p < 0.01; ****p < 0.0001; ##*p* < 0.01; ####*p* < 0.0001.

In light of our ToF-SIMS results, we reasoned that phospholipid remodeling could be affected in silica-NP-exposed cells. Lands cycle is a major remodeling pathway in mammalian cells whereby phospholipids are remodeled after their de novo synthesis.^54^ In this diacylation and reacylation pathway, fatty acyl chains at the *sn*-2 position of PC are hydrolyzed by phospholipases A_2_ (PLA_2_) resulting in the formation of lysophosphatidylcholine (LPC). Unlike cytosolic PLA_2_ (cPLA_2_), calcium-independent phospholipase A_2_ (iPLA_2_) does not require calcium to bind membranes. In particular, the group VIA phospholipase A_2_ (iPLA_2_-VIA) (also known as iPLA_2_β) has been shown to play a major role in phospholipid remodeling in macrophages.^55,56^ We reasoned that LPC could act as an intracellular mediator of pro-inflammatory IL-1β secretion in nonprimed monocytes. Indeed, we found that uncoated silica NPs triggered a significant increase in LPC content in THP-1 cells, which was blocked by bromoenol lactone (BEL), a selective inhibitor of iPLA_2_-VIA (Figure 7a). Moreover, BEL blocked silica NP-induced IL-1β secretion, pointing to a probable role for iPLA_2_-VIA (Figure 7b). BEL was also applied to (nonprimed) primary human CD14+ monocytes, and silica NP-induced IL-1β production was significantly suppressed (Figure 7c). Moreover, BEL partially protected the cells from silica-NP-induced cell death (Figure S20a). Others have recently reported that iPLA2β-mediated detoxification of peroxidized lipids suppresses ferroptosis.^57,58^ However, unlike pyroptosis, which is characterized by IL-1β secretion, pro-inflammatory cytokine secretion has not been explored in ferroptosis. We found that THP-1 cells are susceptible to the classical ferroptosis agonist RSL3 (an inhibitor of GPX4), yet RSL3 failed to trigger IL-1β release (data not shown). Therefore, even though silica NP-induced cell death is ferroptosis-like, it is not identical to ferroptosis.

The inhibitor BEL has been shown to possess >1000-fold selectivity for calcium-independent versus calcium-dependent phospholipase A_2_. However, other enzymatic activities could potentially be affected by BEL.^59^ Therefore, to confirm the role of iPLA_2_-VIA, we silenced *PLA2G6* expression using specific, small interfering RNAs. The reduction of iPLA_2_-VIA expression was confirmed by Western blot (Figure 7d). Silencing of *PLA2G6* yielded a significant reduction in the level of silica NP-triggered IL-1β secretion (Figure 7e). Thus, we propose that lipid peroxidation and the subsequent activation of iPLA_2_-VIA (an enzyme that is involved in plasma membrane repair) are required for amorphous silica NP-triggered IL-1β release in monocytes. Furthermore, we posit that LPC may act as a cell autonomous signal for inflammasome activation in lieu of LPS, meaning that certain silica NPs may license the inflammasome in monocytes in the absence of priming with a microbial ligand. Interestingly, LPC (in oxidized low-density lipoprotein) has previously been shown to stimulate the production of IL-1β in monocytes.^60^ However, in the present study, we provide evidence for a cell autonomous role of LPC upon exposure to silica NPs.

### Evidence for Localized Inflammasome Activation in Silica NP-Exposed Cells

Finally, it is necessary to ask how caspase-dependent IL-1β release can be reconciled with caspase-independent cell death. In a recent study, inflammasome activation was suggested to occur in a single supramolecular structure that coincides with the microtubule-organizing center (MTOC).^61^ To determine whether silica-NP-induced inflammasome activation triggers the formation of such puncta, we exposed THP-1 cells to silica NPs (2.5 μg/mL) and stained the cells using specific antibodies against the inflammasome component, ASC (apoptosis-associated speck-like protein containing a caspase activation and recruitment domain), and the centrosomal marker, γ-tubulin (GTU). We found that inflammasome assembly occurred in a single punctum in the cell (Figure 7f). We then asked whether the assembly of the NLRP3 inflammasome in a single “speck” in silica-NP-exposed cells was subject to regulation by iPLA_2_-VIA. To this end, we visualized the inflammasome (ASC) and the MTOC (GTU) in silica-NP-exposed cells and could show that the colocalization of the two proteins was blocked by BEL (Figure S20b). Further studies are needed to confirm this model of compartmentalized caspase activation. Nevertheless, on the basis of these findings, we propose a model whereby small, uncoated silica NPs trigger restricted (spatially restrained) inflammasome activation in the face of generalized lipid peroxidation-driven cell death. This contrasts with microbial infection where lipid peroxidation was shown to drive a caspase-dependent form of cell death (pyroptosis) in macrophages.^62^ Overall, our studies have shown that lipid peroxidation plays a key role in monocytes exposed to uncoated silica NPs as Trolox was shown to prevent cell death as well as IL-1β production. Notably, Trolox also prevented the accumulation of lipid droplets, and we suggest that cytoplasmic lipid droplet formation may be viewed as a cytoprotective response to the harmful effects of oxidized lipids.

## Conclusions

**Figure 8:**
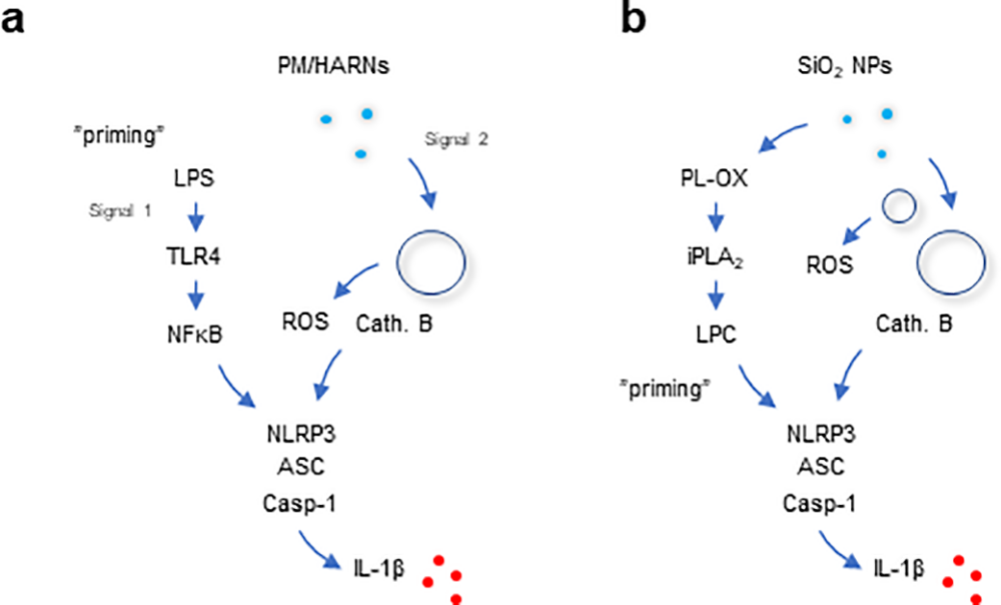
Understanding sterile inflammation. Schematic of the classical two-signal model of inflammasome activation (a) versus the present model (b). The NLRP3 inflammasome consisting of NLRP3, ASC, and pro-caspase-1 serves as a platform for the activation of caspase-1, leading to the proteolysis of pro-IL-1β and the release of IL-1β. Numerous studies have shown that particulate matter (PM) such as alum and crystalline silica (quartz) as well as high aspect ratio (nano)materials (HARNs), e.g., asbestos and carbon nanotubes, are able to trigger the activation of the NLRP3 inflammasome (panel a). This usually occurs through the internalization of the offending agent into phagosomes that eventually fuse with lysosomes (the phago-lysosomal system is depicted here as an empty circle), leading to the generation of reactive oxygen species (ROS) and the release of lysosomal cathepsin B. Macrophages are usually primed with a Toll-like receptor (TLR) agonist such as LPS (lipopolysaccharide). In contrast, we show that uncoated amorphous silica NPs are capable of triggering NLRP3-dependent IL-1β release in the absence of LPS priming (panel b) and we provide evidence that mitochondrial ROS production may contribute to the effects of silica NPs on membrane lipids (mitochondria are depicted here as a small empty circle). We posit that lipid peroxidation, leading to phospholipase (iPLA_2_) activation and the generation of lysophosphatidylcholine (LPC), provides a cell autonomous “priming” signal.

Using a mass-spectrometry-based approach, we have shown that amorphous silica NPs trigger inflammasome activation in monocytes in the absence of a microbial priming signal. Using ToF-SIMS, we provided molecular evidence of specific changes in plasma membrane lipids in cells exposed to uncoated silica NPs. We could show that silane modification of silica NPs abolished cellular responses. The activation of iPLA_2_-VIA (iPLA_2_β) with the generation of LPC is a secondary event related to phospholipid remodeling.^54^ Hence, according to our current model, lipid signaling links NP-triggered membrane damage to cytokine release, manifested as inflammasome activation. Furthermore, inflammasome-dependent production of IL-1β was accompanied by a non-apoptotic cell death associated with lipid peroxidation, which we refer to as *lipoptosis*, and we submit that lipoptosis may be viewed as a noncanonical form of ferroptosis. These findings shed light on the mechanism of silica-NP-induced inflammasome activation (Figure 8) and the lipid peroxidation-dependent signaling cascade in sterile inflammation.

## Experimental Section

### Silica Particle Synthesis and Characterization

Colloidal silica nanoparticles (NPs) (Table S1) were obtained from Nouryon (Bohus, Sweden). The stock suspensions of the NPs were stabilized by using an anionic sodium (Na) solution. The silane-modified SiO_2_ NPs were modified by reacting alkyltrialkoxysilanes with the silanol groups, resulting in steric stabilization of the NPs with glycerol–propyl tails, while the Al-SiO_2_ NPs were modified using Na–aluminate such that aluminate ions Al(OH)_4_^–^ were incorporated in the surface. The degree of surface modification was <10%. Silica NPs without surface modification are referred to as uncoated. FITC-labeled SiO_2_ NPs were prepared using a modified Stöber synthesis (Table S1). In brief, a fluorescent precursor was prepared by reacting fluorescein isothiocyanate (FITC) with (3-aminopropyl)trimethoxysilane. This conjugate was then condensed with tetraethyl orthosilicate (TEOS) to yield fluorescent particles in a mixture of water, ethanol, and ammonia. The particles were purified and further coated with an extra layer of silica. Two “benchmark” NPs produced by the precipitation (NM200) and pyrolysis methods (NM203) (Table S1) were also included. These NPs were procured from the nanomaterial repository of the Joint Research Centre (JRC). Fresh dispersions were prepared for each experiment, as described previously.^45^ For long-term aging, dispersions of NM200 and NM203 were stored at 4 °C for 18 months.

#### TEM/SEM

NP samples were examined using a Jeol 2000FXII transmission electron microscope operated at 160 kV. Micrographs were obtained with a Megaview-III digital camera. TEM images of ≥50 NPs per sample were scored to determine the size distribution. SEM micrographs were obtained using an LEO 55 Ultra field emission SEM.

#### AFM

The surface structure of the samples was investigated using a Bruker FastScan Bio atomic force microscope (AFM) in scan assist mode. Samples were deposited onto magnetic holders covered by carbon tape and air-dried overnight prior to investigation.

#### EDS

Elemental mapping of the samples was obtained on an environmental scanning electron microscope Hitachi TM-1000-μ-DeX coupled with an Oxford Instrument EDS detector. The samples were mounted on an aluminum stage coated by carbon tape tabs.

#### FTIR

Spectra of all samples were recorded as KBr pellets on a PerkinElmer Spectrum 100 instrument. A total of eight scans were carried out on wavenumbers from 400 to 4000 cm^–1^, in transmittance mode. All spectra were smoothed and baseline-corrected.

#### TGA

TGA was performed on a Pyris 1 thermobalance instrument in an ambient atmosphere.

#### EPR

The silica NPs were prepared for electron paramagnetic resonance (EPR) measurements as described by Lehman et al.^63^ Briefly, the reaction was set by mixing silica NPs (5 wt %) and H_2_O_2_ (200 mM). The spin-trap, 5,5-dimethyl-1-pyrroline *N*-oxide (DMPO; 25 mM), was added in the reaction mixture to bind ^•^OH radicals due to their short half-life. The samples were equilibrated for 15 min at room temperature before analysis. EPR measurements were performed using a Bruker Elexsys E500 X-band spectrometer operating at 9.75 GHz equipped with a standard TE102 flat cell from Wilmad providing a sample volume of 150 μL. The EPR spectra were recorded at room temperature, and 5,5-dimethyl-1- pyrroline *N*-oxide (DMPO) was used as a spin trap. The spin-Hamiltonian parameters used to fit the two DMPO adducts were *a*_N_ = 14.2 G, *a*_H_^β^ = 11.4 G, and *a*_H_^γ1^ = 1.2 G for the DMPO-^•^OOH adduct and *a*_N_ = *a*_H_^β^ = 14.9 G for the DMPO-^•^OH adduct. These parameters are consistent with the ones reported in the literature.^63^

#### DLS

Hydrodynamic size and ζ potential were determined by dynamic light scattering (DLS) and phase analysis light scattering analysis using the Malvern Zetasizer nano ZS, equipped with a 633 nm laser. The NP samples were prepared by diluting stock solution in water and cell medium at 50 μg/mL and the analysis was performed immediately after dispersion.

#### Endotoxin Content

The silica NPs were evaluated for endotoxin content by using the standard chromogenic Limulus amoebocyte lysate (LAL) assay (Lonza, Walkersville, MD, USA), as described.^45^ The test materials were all found to be endotoxin-free (data not shown).

### Single-Cell Mass Cytometry (Cytometry by Time-of-Flight)

Single-cell mass cytometry analysis was performed as described previously.^16^ PBMCs, obtained from healthy adult donors, were separated from ethylenediamine tetraacetic acid (EDTA)–venous blood using a Ficoll–Paque (GE Healthcare) standard isolation protocol. All of the experiments were carried out in accordance with institutional guidelines. PBMCs were maintained in RPMI-1640 medium supplemented with 10% FBS. PBMCs were seeded at a concentration of 4.0 × 10^6^ cells per well (6-well plates) and exposed to 0.1 μg/mL bare SiO_2_ or ES-SiO_2_ NPs for 24 h. LPS (0.1 μg/mL; Sigma-Aldrich) and untreated cells were used as positive and negative controls, respectively. Six hours before the end of the treatment, cells were incubated with Brefeldin A (Invitrogen) at a final concentration of 10 μg/mL. Following the exposure, cells were stained with Cell-ID Intercalator-103Rh (Fluidigm, South San Francisco, CA, USA) 1:500 for 15 min at 37 °C, subsequently washed and combined using Cell-ID 20-Plex Pd Barcoding Kit (Fluidigm), and stained with Maxpar Human Peripheral Blood Phenotyping and Human Intracellular Cytokine I Panel Kits (Fluidigm) following the manufacturer’s protocol. The antibody cocktail was added at a dilution of 1:100 for each antibody. After incubation, cells were incubated with a Cell-ID Intercalator-Ir solution at a final concentration of 125 nM for 5 min. Rh103 is a cationic nucleic-acid-intercalating molecule that is used for the discrimination of dead cells from live cells. Before data acquisition, each sample was filtered with a 0.22 μm cell strainer cap to remove possible cell clusters or aggregates. Data were analyzed using the mass cytometry platform CyToF2 (Fluidigm). The data analysis was performed according to Orecchioni et al.^16^ Specific subpopulations were defined as reported in Figure S5. In brief, the following markers were used: T-cells (CD45+ CD19– CD3+), T helper (CD45+ CD3+ CD4+), T-cytotoxic (CD45+ CD3+ CD8+), T naive (CD45RA+ CD27+ CD38– HLA-DR−), T-effector (CD45RA+ CD27– CD38– HLA-DR−), activated T-cells (CD38+ HLA-DR+), B-cells (CD45+ CD3– CD19+), B-naive (HLA-DR+ CD27−), B-memory (HLA-DR+ CD27+), plasma B (HLA-DR– CD38+), NK cells (CD45+ CD3– CD19– CD20– CD14– HLA-DR– CD38+ CD16+), classical monocytes (CD45+ CD3– CD19– CD20– HLA-DR+ CD14dim CD16+), nonclassical monocytes (CD45+ CD3– CD19– CD20– HLA-DR+ CD14+ CD16+), myeloid DCs (CD45+ CD3– CD19– CD20– CD20– CD14– HLA-DR+ CD11c+ CD123−), plasmacytoid DCs (CD45+ CD3– CD19– CD20– CD14– HLA-DR+ CD11c– CD123+). The resulting data were visualized using the viSNE tool.^18^ The following cell surface markers were used to draw the maps: CD3, CD4, CD8a, CD11c, CD14, CD16, CD19, CD20, CD123, and HLA-DR. The cytokine data obtained by single-cell analysis were also analyzed using the viSNE tool. Heat maps of the mean marker expression ratios of the indicated cytokines are shown.

### Cell Lines and Primary Human Monocytes

The human monocyte-like cell line THP-1 (American Type Culture Collection) was maintained in RPMI-1640 medium supplemented with 10% heat-inactivated FBS (Sigma), 2 mM glutamine (Gibco), penicillin (100 U/mL), and streptomycin (100 μg/mL). LPS priming was performed in some experiments by stimulating the cells with 0.1 μg/mL LPS for 2 h. For differentiation into macrophage-like cells, THP-1 cells were stimulated with PMA (200 nM) for 3 days. Primary human CD14-positive monocytes were isolated from buffy coats obtained from adult blood donors (Karolinska University Hospital, Stockholm). Monocytes were separated using CD14 MicroBeads (Miltenyi Biotec Ltd.).^47^ The cells were maintained in an RPMI-1640 medium with 10% FBS, 2 mM glutamine, 100 U/mL penicillin, and 100 μg/mL streptomycin. The identity of the human blood donors is unknown to the investigators performing the experiments, and the data cannot be traced back to the individual donors.

### Primary Human Natural Killer Cell Isolation

Buffy coats from anonymous donors were obtained from the Karolinska University Hospital, Stockholm (see above). NK cells were obtained through negative selection using the NK cell isolation kit (Miltenyi Biotec Ltd.). In brief, peripheral blood mononuclear cells (PBMCs) were isolated by Ficoll–Hypaque gradient separation (GE-Healthcare), followed by incubation with biotinylated antibody cocktail (Miltenyi Biotec Ltd.) at 4 °C. Antibiotin beads were then added for a further 15–20 min, and the cells were washed and added to magnetic columns. The purity of the enriched polyclonal NK cells was measured as percentage of the CD56^+^ (BioLegend Cat. No. 317343) CD3^–^ (BioLegend Cat. No. 362504) population using a CyAN ADP LX 9-color flow cytometer (Beckman Coulter). Data were analyzed by using FlowJo software.

### Inflammasome Knockout Cell Lines

The wild-type THP-1 cell line (designated Null-1), and the NLRP3-deficient (defNLRP3) and caspase-1-deficient (defCASP1) cell lines, were obtained from InvivoGen (Toulouse, France). The cells were cultured in DMEM supplemented with 10% FBS, 100 U/mL penicillin, 100 μg/mL streptomycin, glutamine (2 mM), and 1X HEK-Blue selection antibiotics mixture (InvivoGen) as previously described.^44^ NP experiments were performed using nonprimed, nondifferentiated cells. For experiments using nigericin (Sigma-Aldrich), cells were either primed or nonprimed.

### Cell Viability Assessment

#### Alamar Blue Assay

Loss of cell viability was determined using the Alamar blue assay (Thermo Fisher Scientific), as described previously.^45^ Experiments were performed with primary monocytes, NK cells, and THP-1 cells. The results were derived from three independent experiments (independent donors), each performed in triplicate. To explore the mechanism of toxicity, THP-1 cells were incubated with inhibitors of apoptosis (the pan-caspase inhibitor, zVAD-fmk), necroptosis (the RIP1 kinase inhibitor, necrostatin-1), ferroptosis (the iron-chelating compound, DFO, and the radical scavenging compound ferrostatin-1), and lipid peroxidation (the water-soluble lipid antioxidant, Trolox, 6-hydroxy-2,5,7,8-tetramethylchroman-2-carboxylic acid; Sigma). Cells were pre-incubated with the inhibitors for 30 min and then exposed to silica NPs. To trigger ferroptosis, cells were exposed to RSL3 (CAS Reg. No. 1219810-16-8; Sigma-Aldrich).

#### LDH Release Assay

LDH release was measured to corroborate the cell viability results. To this end, THP-1 cells were exposed to the silica NPs for 12 h; in some experiments, cells were pre-incubated with the indicated pharmacological inhibitors for 30 min. After exposure, supernatants were collected and processed for LDH release measurement using the CytoTox 96 Non-Radioactive Cytotoxicity Assay kit (Promega). The samples were analyzed by using a Tecan Infinite F200 spectrophotometer (Männedorf, Switzerland). Results are expressed as percent LDH release versus the maximum LDH release (cell lysis).

### Nanoparticle Uptake and Subcellular Distribution

#### Flow Cytometry

The cellular association of colloidal silica in THP-1 cells was quantified by the detection of FITC-labeled NPs. THP-1 cells cultured in RPMI-1640 medium supplemented with 10% FBS were exposed to FITC-labeled NPs at final concentrations of 5 and 10 μg/mL for 1 and 6 h. Cells were then washed three times, resuspended in PBS, and analyzed using a BD LSRFortessa flow cytometer operating with BD FACS DIVA software (BD Biosciences). The fluorescence intensity of silica NPs alone (without cells) was measured as a control. Gating was applied to avoid any interference of residual colloidal silica that had not been taken up by cells. Data were analyzed by using FCS Express 4 Flow Cytometry software. For some experiments, cells were preincubated with 100 μg/mL fucoidan (Sigma) for 30 min before the addition of the NPs. The colloidal NPs were then added directly to the well, together with the inhibitors, and were mixed by careful pipetting.

#### Confocal Microscopy

THP-1 (1 × 10^6^ cells/mL) was seeded into a 24-well plate containing poly-l-lysine (Sigma) coated coverslips. Cells were allowed to attach for 1 h and then washed with PBS. Then, cells were exposed to FITC-labeled silica NPs at 50 μg/mL for 6 h. Counterstaining with 5 μg/mL of phalloidin-TRITC (Sigma) was done after fixation of cells with 4% formaldehyde. The coverslips were mounted on glass slides with VECTASHIELD antifade mounting medium with DAPI (Invitrogen) and a Zeiss LSM880 confocal microscope was for visualization. Data were analyzed using ZEN software (Zeiss).

#### TEM

THP-1 cells maintained in complete cell culture medium at 1.0 × 10^6^ cells per well were incubated with 10 μg/mL silica NPs for 2 and 12 h. TEM samples were processed according to the procedure described previously.^64^ In brief, at the end of the incubation time, cells were washed thrice with serum-free medium and fixed with 4% glutaraldehyde in 0.1 M sodium phosphate buffer, pH 7.4 for 1 h at 4 °C. Following postfixation in 1% OsO_4_ in 0.1 sodium phosphate buffer for 1 h at 4 °C, the samples were serially dehydrated in a gradient of ethanol followed by acetone and LX-112 infiltration and finally embedded in LX-112. Ultrathin sections (approximately 50–80 nm) were prepared using a Leica EM UC6, contrasted with uranyl acetate followed by lead citrate, and examined in Hitachi HT 7700 electron microscope (Hitachi High-Technologies). Digital images were acquired using a 2kx2k Veleta CCD camera (Olympus Soft Imaging Solutions).

### Nanoscale Secondary Ion Mass Spectrometry

For label-free detection of the silica NPs, we employed nanoSIMS, as described.^23^ To this end, the grids used for TEM imaging were subsequently used for nanoSIMS imaging. The ion images were acquired using a Cameca NanoSIMS 50LnanoSIMS instrument (Genevilliers, France). Measurements were performed by rastering the sample surface with a 16 keV Cs^+^ primary ion beam. The measurements were performed with a 1 pA (aperture diaphragm D13) primary current. The spatial resolution of the primary beam size was 200 nm. The ^12^C_2_^–^, ^12^C^14^N^–^, and ^28^Si^–^ species were measured simultaneously in multicollection mode using electron multipliers. Mass-filtered images of the cells were acquired using entrance slit 2 (ES2; width = 25 μm) and aperture slit 3 (AS3; width = 350 μm). The energy slit was kept fully open (energy band pass up to 100 eV). The relative transmission of the mass spectrometer is ∼70% with a mass resolving power of 6,500 on ^28^Si^–^. Each area was implanted with a fluence of 10^17^ Cs^+^.cm^–2^ prior to measurement. Imaged areas ranged from 13 μm × 13 to 20 μm × 20 μm raster size, sampled by 256 × 256 pixels with a dwell time of 5 ms per pixel. Images are stacks of 5–20 planes (for each measured mass). Images were processed with WinImage Cameca SIMS software. The count rates were corrected using a dead time of 44 ns for each electron multiplier. The ^28^Si^–^/^12^C_2_^–^ of the surrounding epoxy resin of each sample was in the range of 8 × 10^–6^ to 6 × 10^–5^.

### Time-of-Flight Secondary Ion Mass Spectrometry

Cells were prepared for ToF-SIMS analysis as described before.^35^ Briefly, THP-1 cells (5.0 × 10^4^) were seeded on sterile plastic coverslips (Thermo Fisher Scientific) after coating with poly-l-lysine. The cells were then exposed for 2 and 6 h to the bare and silane-modified silica NPs at a concentration of 2.5 μg/mL, with and without pre-incubation with the lipid antioxidant Trolox (500 μM) for 30 min. Thereafter, the cells were gently washed with ammonium acetate for 30 s and then air-dried for 24 h in a laminar flow hood. ToF-SIMS analysis was performed using a TOF.SIMS 5 instrument (ION-TOF GmbH, Münster, Germany) equipped with a 25 keV Bi cluster ion gun as the primary ion source and a 10 keV C_60_ ion source for sputtering and depth profiling. The samples were analyzed using a pulsed primary ion beam (Bi_3_^+2^, 0.34 pA at 50 keV) with a focus of approximately 2 μm using the high current bunched mode to obtain high mass resolution spectra. For higher lateral resolution imaging the delayed extraction mode^65^ was used (Bi_3_^+2^, 0.2 pA at 50 keV) with a focus of approximately 400 nm. The mass resolution using this setup was at least *M*/Δ*M* = 3000 fwhm at *m*/*z* 500. All spectra were acquired and processed with Surface Lab software version 6.4 (ION-TOF GmbH). The spectra were internally calibrated to signals of [C]^+^, [CH_2_]^+^, [CH_3_]^+^, [C_5_H_15_PNO_4_]^+^, and [C_27_H_45_]^+^ for the positive ion mode and [C]^−^, [CH]^−^, [C_2_], [C_3_]^−^ for the negative ion mode. Depth profile analysis was performed using a C_60_^+2^ beam at 20 keV with a current of 0.2 nA in a non-interlaced mode with 1 s of analysis, 1 s of sputtering, and a pause of 1 s for each sputter cycle at rasters of 200 μm × 200 to 350 μm × 350 μm, corresponding to 2× the imaged area. The maximum ion dose density of Bi_3_^+2^ was kept between 1 × 10^12^ and 2 × 10^12^ cm^–2^ over the whole depth profiling experiment, while the ion dose for C_60_^+2^ ranged from 1 × 10^14^ to 4 × 10^14^ ions per cm^2^. Low-energy electrons were used for charge compensation during the entire analysis. The Surface Lab software (version 6.3 ION-ToF, GmbH, Germany) was used to analyze the data.^35^ The complete data set was normalized to the primary ion dose of the respective spectra and analyzed by PCA and the multivariate data analysis program SIMCA version 13.0.

### Inflammasome Activation and IL-1β Detection

THP-1 cells were exposed to uncoated or surface-modified (Al and ES) NPs (2.5 μg/mL) for 6 h, and samples were collected and stored at −80 °C. IL-1β release was determined using a human IL-1β ELISA kit (Invitrogen, Sweden) according to the manufacturer’s instruction. Absorbance was measured at 450 nm using a Tecan Infinite F200 plate reader. Results are expressed as pg/50.000 cells of released cytokine, based on at least three independent experiments. For experiments using different inhibitors, cells were pre-incubated for 1 h with the indicated inhibitors, z-VAD-fmk (20 μM, R&D Systems), CaO74Me (10 μM, Sigma-Aldrich), A438079 (50 μM, Sigma-Aldrich), Trolox (500 μM, Sigma-Aldrich), and MCC950 (10 μM, Sigma-Aldrich). Then, cells were exposed to uncoated or surface-modified (Al and ES) NPs, cell media were collected, and IL-1β quantification was performed as described above. To validate the role of NLRP3, the Null-1 and defNLRP3 THP-1 cell lines were used.^47^ The cells were grown with selection antibiotics (see: “inflammasome knockout cells”). Cells were plated at a density of 10^5^ cells/well and exposed for 6 h to bare and surface-modified silica NPs (2.5 μg/mL) in medium supplemented with 10% FBS. Cell media were collected, and IL-1β content was measured using a specific ELISA. Additionally, TNF-α was determined using the TNF alpha ELISA kit (Invitrogen, Sweden).

### Immunocytochemistry

THP-1 cells were seeded overnight at a density of 1.8 × 10^5^ cells/cm^2^ on a poly-l-lysine-coated coverslip in a 24-well plate. The next day, cells were exposed to 2.5 μg/mL bare silica NPs. Then, coverslips were washed three times in PBS and permeabilized in 0.1% Triton-X 100 (Sigma-Aldrich) for 15 min, followed by blocking with 10% goat serum (Abcam) and 0.1% Triton-X100 for 1 h. Next, coverslips were incubated with mouse anti-ASC antibody (Santa Cruz Biotechnology) or rabbit anti-GTU antibody (Abcam) in antibody buffer (8% goat serum and 0.1% Triton-X-100) overnight at 4 °C. Coverslips were then rinsed in PBS and incubated with Alexa Fluor 488 conjugated goat antimouse antibody and Alexa Fluor 595 conjugated goat antirabbit antibody (both from Life Technologies, Thermo Fisher Scientific) for 1 h. Coverslips were mounted on glass slides using DAPI-containing mounting medium (Invitrogen) and imaged using a Zeiss LSM880 confocal microscope and ZEN software (Zeiss). For the visualization of lipid droplets, Nile Red (CAS 7385-67-3) Sigma-Aldrich) was added at 0.5 μg/mL for 15 min at the end of the cell incubation. Cells were counterstained with DAPI for visualization of cell nuclei, and samples were imaged as above.

### Cellular GSH Content

THP-1 cells were pre-incubated with or without Trolox (500 μM) for 30 min and exposed to the indicated concentrations of silica NPs for 6 h. After exposure, the plates were centrifuged, supernatants were discarded, and the samples were analyzed by using the GSH-Glow assay (Promega) (65). GSH-Glow reagent was added, and cells were incubated for 30 min followed by the addition of luciferin detection reagent. Luminescence was measured using the Tecan Infinite 200 plate reader.

### Cellular ROS Production

#### Total Cellular ROS

THP-1 cells were loaded with 10 μM DCF-DA (Invitrogen) and incubated for 30 min in the dark at 37 °C. Following incubation, the cells were washed twice and suspended in fresh cell culture medium. DCF-DA-loaded cells were then exposed to the uncoated or silane-modified SiO_2_ NPs for 1 h. After exposure, DCF fluorescence was recorded using a BD LSRFortessa flow cytometer operated with BD FACS-DIVA software (BD Biosciences).

#### Mitochondrial ROS

The production of mitochondrial O_2_^•–^ was quantified using the MitoSOX assay (Invitrogen) according to the manufacturer’s instruction. Briefly, THP-1 cells were exposed to silica NPs at the indicated concentrations. After exposure, cells were collected and stained with MitoSOX dye for 30 min. Cells were then washed and analyzed by flow cytometry, as above. To confirm the role of mitochondrial ROS, MitoTEMPO (10 μM) was used (Sigma-Aldrich).

### Lipid Peroxidation and Lipid Droplet Content

Lipid peroxidation was determined after the cells were exposed to silica NPs for 6 h. Cells were pre-incubated as indicated with 500 μM Trolox (Sigma) for 1 h at 37 °C prior to addition of the NPs. Additionally, for some experiments, cells were pre-incubated for 1 h with the DGAT-1 inhibitor A 922500 (Sigma-Aldrich) and/or the DGAT-2 inhibitor PF-06424439 (Sigma-Aldrich) at 5 μM. The cells were collected and resuspended in PBS containing 2 μM C11-BODIPY 581/591 (Thermo Fisher Scientific). The cells were then incubated for 30 min in the dark at 37 °C, and the analysis was performed using the BD LSRFortessa flow cytometer operating with BD FACS-DIVA software (BD Biosciences). To evaluate the content of lipid droplets, cells were incubated with BODIPY 493/503 (Thermo Fisher Scientific) for 15 min at 37 °C. Then, cells were harvested and analyzed by using the BD LSRFortessa flow cytometer.

### Western Blotting

For protein detection, 1.0 × 10^6^ cells were seeded and exposed to silica NPs at 2.5 μg/mL with or without pre-incubation with the specified inhibitors. The cells were then collected and lysed overnight at 4 °C in RIPA buffer [50 mM Tris HCl (pH 7.4), 150 mM NaCl, 1% Triton X-100, 0.25% sodium deoxycholate, 0.1% SDS, and 1 mM EDTA]. Protease and phosphatase inhibitors (Mini EDTA-free Protease Inhibitor Cocktail, Sigma-Aldrich; 1 mM PMSF, Thermo Fisher; PhosSTOP, Sigma-Aldrich) and 1 mM DTT (Sigma-Aldrich) were freshly added to the buffer. Cell lysates were centrifuged at 13.000*g* for 20 min, and supernatants were collected. The protein concentration was measured by Bradford assay, and 30–50 μg of protein was loaded into each well of a NuPAGE 4–12% Bis-Tris gradient gel (Thermo Fisher) and subjected to electrophoretic separation. The proteins were then transferred to a Hybond Low-Fluorescent 0.2 μm PVDF membrane (Amersham), blocked for 1 h in Odyssey Blocking Buffer (PBS; LI-COR), and stained overnight at 4 °C with primary antibodies against iPLA_2_ (Sigma-Aldrich, SAB4200130). Membranes were reprobed for GAPDH (Thermo Fisher). The goat antirabbit IgG (H+L) HRP-conjugated antibody (Thermo Fisher Scientific) or goat antimouse IRDye 680RD antibody (LI-COR) was used as a secondary antibody. The proteins were analyzed on the LI-COR Odyssey CLx scanner using Odyssey Image Studio software.

### Silencing of *PLA2G6*

*PLA2G6* expression was silenced using a specific human siRNA oligo duplex (Locus ID 8398, cat. no. SR305501) that was obtained from OriGene Technologies, Inc. (Rockville, MD, USA). siRNA transfection was performed using the Amaxa cell line nucleofector kit V obtained from Lonza (Basel, Switzerland). Briefly, 1 million THP-1 cells were transfected with 100 nM *PLA2G6*-siRNA using the Nucleofector device (Lonza). The cells were transferred immediately into 12-well plates and allowed to grow for 48 h in RPMI-1640 cell medium supplemented with 10% FBS and 2 mM l-glutamine without adding the antibiotics. Cell viability after 48 h of transfection was determined using the Trypan blue assay. The successful silencing of *PLA2G6* was verified by Western blot analysis of iPLA_2_-VIA, as described in the preceding section.

### Cellular LPC content

The intracellular LPC content was measured in THP-1 cells after 6 h of exposure to bare silica NPs (2.5 μg/mL). Lipid isolation and LPC measurements were performed by using the enzymatic LPC assay obtained from BioVision, Inc. (Milpitas, CA, USA). Briefly, 2 million cells were collected from each sample. The cells were homogenized, and the lipid extraction was performed in chloroform:methanol (1:2) solvent. The solvent was then evaporated, and the isolated lipids were dissolved in a lipid resuspension buffer. LPC content was determined using a Tecan Infinite F200 plate reader.

### Statistics

Experiments were performed at least thrice. Data are shown as mean values ± SD. Statistical tests were performed using ANOVA followed by Dunnett’s and/or Tukey’s post hoc analysis. The significance limits are described in the respective figure legends.

## Supporting Information Available

Schematic study design; table summarizing the physicochemical properties of silica nanoparticles; TEM, SEM, EDS, TGA, and AFM of the silica nanoparticles; EPR results; gating strategy used for single-cell mass cytometry; cytokine results at single-cell level; cell viability and cytokine results for natural killer (NK) cells; TEM, confocal microscopy, and flow cytometry results showing cellular uptake of labeled and non-labeled silica nanoparticles; confocal microscopy and flow cytometry results on lipid droplet formation; cell viability assessment of fresh *versus* aged silica nanoparticles; lipid peroxidation, cellular GSH content, and cell viability/metabolic capacity with/without various cell death inhibitors; ToF-SIMS imaging results, and principal component analysis (PCA) of ToF-SIMS data; TNF-α production in primary human monocytes and monocyte-like THP-1 cells; IL-1β production in response to a panel of silica nanoparticles, and IL-1β production in monocyte-like *versus* macrophage-like THP-1 cells in the presence or absence of LPS priming; IL-1β production in wild-type *versus* caspase-1-deficient THP-1 cells, and impact of nigericin; ROS production in response to uncoated *versus* silane-modified silica particles; impact of BEL and MitoTEMPO on cell viability, and co-localization of ASC and GTU (PDF)

## Abbreviations

ASC: apoptosis-associated speck-like protein containing a caspase activation and recruitment domain
BEL: bromoenol lactone
CyToF: cytometry by time-of-flight
EPR: electron paramagnetic resonance
GSH: glutathione
LPC: lysophosphatidylcholine
LPS: lipopolysaccharide
MTOC: microtubule-organizing center
NLRP3: NLR family pyrin domain containing 3 (NLR, nucleotide binding domain (NOD)-like receptor)
NPs: nanoparticles
nanoSIMS: nanoscale secondary ion mass spectrometry
PBMCs: peripheral blood mononuclear cells
PCA: principal component analysis
PLA_2_: phospholipase A_2_ (“c” for cytosolic, “i” for calcium-independent)
RSL3: *Ras*-selective lethal small molecule 3
ToF-SIMS: time-of-flight secondary ion mass spectrometry
TEM/SEM: transmission/scanning electron microscopy
TLR: Toll-like receptor
t-SNE: t-distributed stochastic neighbor embedding (viSNE is a visualization tool based on t-SNE)

## References

[ref1] Broz P.; Dixit V. M. Inflammasomes: Mechanism of Assembly, Regulation and Signalling. Nat. Rev. Immunol. 2016, 16, 407–420. 10.1038/nri.2016.58. 27291964

[ref2] Sun B.; Wang X.; Ji Z.; Li R.; Xia T. NLRP3 Inflammasome Activation Induced by Engineered Nanomaterials. Small 2013, 9, 1595–1607. 10.1002/smll.201201962. 23180683 PMC4056676

[ref3] Dostert C.; Pétrilli V.; Van Bruggen R.; Steele C.; Mossman B. T.; Tschopp J. Innate Immune Activation Through Nalp3 Inflammasome Sensing of Asbestos and Silica. Science 2008, 320, 674–677. 10.1126/science.1156995. 18403674 PMC2396588

[ref4] Cassel S. L.; Eisenbarth S. C.; Iyer S. S.; Sadler J. J.; Colegio O. R.; Tephly L. A.; Carter A. B.; Rothman P. B.; Flavell R. A.; Sutterwala F. S. The Nalp3 Inflammasome is Essential for the Development of Silicosis. Proc. Natl. Acad. Sci. U. S. A. 2008, 105, 9035–9040. 10.1073/pnas.0803933105. 18577586 PMC2449360

[ref5] Hornung V.; Bauernfeind F.; Halle A.; Samstad E. O.; Kono H.; Rock K. L.; Fitzgerald K. A.; Latz E. Silica Crystals and Aluminum Salts Activate the NALP3 Inflammasome Through Phagosomal Destabilization. Nat. Immunol. 2008, 9, 847–856. 10.1038/ni.1631. 18604214 PMC2834784

[ref6] Morishige T.; Yoshioka Y.; Inakura H.; Tanabe A.; Yao X.; Narimatsu S.; Monobe Y.; Imazawa T.; Tsunoda S.; Tsutsumi Y.; Mukai Y.; Okada N.; Nakagawa S. The Effect of Surface Modification of Amorphous Silica Particles on NLRP3 Inflammasome Mediated IL-1β Production, ROS Production and Endosomal Rupture. Biomaterials 2010, 31, 6833–6842. 10.1016/j.biomaterials.2010.05.036. 20561679

[ref7] Yazdi A. S.; Guarda G.; Riteau N.; Drexler S. K.; Tardivel A.; Couillin I.; Tschopp J. Nanoparticles Activate the NLR Pyrin Domain Containing 3 (Nlrp3) Inflammasome and Cause Pulmonary Inflammation Through Release of IL-1α and IL-1β. Proc. Natl. Acad. Sci. U. S. A. 2010, 107, 19449–19454. 10.1073/pnas.1008155107. 20974980 PMC2984140

[ref8] Kang L.; Dai J.; Wang Y.; Shi P.; Zou Y.; Pei J.; Tian Y.; Zhang J.; Buranasudja V. C.; Chen J.; Cai H.; Gao X.; Lin Z. Blocking Caspase-1/Gsdmd and Caspase-3/-8/Gsdme Pyroptotic Pathways Rescues Silicosis in Mice. PLoS Genet. 2022, 18, e1010515 10.1371/journal.pgen.1010515. 36459518 PMC9718385

[ref9] Zhang H.; Dunphy D. R.; Jiang X.; Meng H.; Sun B.; Tarn D.; Xue M.; Wang X.; Lin S.; Ji Z.; Li R.; Garcia F. L.; Yang J.; Kirk M. L.; Xia T.; Zink J. I.; Nel A.; Brinker C. J. Processing Pathway Dependence of Amorphous Silica Nanoparticle Toxicity: Colloidal vs Pyrolytic. J. Am. Chem. Soc. 2012, 134, 15790–15804. 10.1021/ja304907c. 22924492 PMC3505689

[ref10] Croissant J. G.; Butler K. S.; Zink J. I.; Brinker C. J. Synthetic Amorphous Silica Nanoparticles: Toxicity, Biomedical and Environmental Implications. Nat. Rev. Mater. 2020, 5, 886–909. 10.1038/s41578-020-0230-0.

[ref11] Shi J.; Karlsson H. L.; Johansson K.; Gogvadze V.; Xiao L.; Li J.; Burks T.; Garcia-Bennett A.; Uheida A.; Muhammed M.; Mathur S.; Morgenstern R.; Kagan V. E.; Fadeel B. Microsomal Glutathione Transferase 1 Protects Against Toxicity Induced by Silica Nanoparticles but not by Zinc Oxide Nanoparticles. ACS Nano 2012, 6, 1925–1938. 10.1021/nn2021056. 22303956 PMC3314313

[ref12] Dixon S. J.; Lemberg K. M.; Lamprecht M. R.; Skouta R.; Zaitsev E. M.; Gleason C. E.; Patel D. N.; Bauer A. J.; Cantley A. M.; Yang W. S.; Morrison B.; Stockwell B. R. Ferroptosis: an Iron-Dependent Form of Nonapoptotic Cell Death. Cell 2012, 149, 1060–1072. 10.1016/j.cell.2012.03.042. 22632970 PMC3367386

[ref13] Tsugita M.; Morimoto N.; Tashiro M.; Kinoshita K.; Nakayama M. SR-B1 is a Silica Receptor That Mediates Canonical Inflammasome Activation. Cell Rep. 2017, 18, 1298–1311. 10.1016/j.celrep.2017.01.004. 28147282

[ref14] Lo Giudice C.; Yang J.; Poncin M. A.; Adumeau L.; Delguste M.; Koehler M.; Evers K.; Dumitru A. C.; Dawson K. A.; Alsteens D. Nanophysical Mapping of Inflammasome Activation by Nanoparticles *via* Specific Cell Surface Recognition Events. ACS Nano 2022, 16, 306–316. 10.1021/acsnano.1c06301. 34957816

[ref15] Chen G. Y.; Nunez G. Sterile Inflammation: Sensing and Reacting to Damage. Nat. Rev. Immunol. 2010, 10, 826–837. 10.1038/nri2873. 21088683 PMC3114424

[ref16] Orecchioni M.; Bedognetti D.; Newman L.; Fuoco C.; Spada F.; Hendrickx W.; Marincola F. M.; Sgarrella F.; Rodrigues A. F.; Ménard-Moyon C.; Cesareni G.; Kostarelos K.; Bianco A.; Delogu L. G. Single-Cell Mass Cytometry and Transcriptome Profiling Reveal the Impact of Graphene on Human Immune Cells. Nat. Commun. 2017, 8, 1109 10.1038/s41467-017-01015-3. 29061960 PMC5653675

[ref17] Yang Y. S.; Atukorale P. U.; Moynihan K. D.; Bekdemir A.; Rakhra K.; Tang L.; Stellacci F.; Irvine D. J. High-Throughput Quantitation of Inorganic Nanoparticle Biodistribution at the Single-Cell Level Using Mass Cytometry. Nat. Commun. 2017, 8, 14069 10.1038/ncomms14069. 28094297 PMC5247578

[ref18] Amir E.-a. D.; Davis K. L; Tadmor M. D; Simonds E. F; Levine J. H; Bendall S. C; Shenfeld D. K; Krishnaswamy S.; Nolan G. P; Pe'er D. viSNE Enables Visualization of High Dimensional Single-Cell Data and Reveals Phenotypic Heterogeneity of Leukemia. Nat. Biotechnol. 2013, 31, 545–552. 10.1038/nbt.2594. 23685480 PMC4076922

[ref19] Vis B.; Hewitt R. E.; Faria N.; Bastos C.; Chappell H.; Pele L.; Jugdaohsingh R.; Kinrade S. D.; Powell J. J. Non-Functionalized Ultrasmall Silica Nanoparticles Directly and Size-Selectively Activate T cells. ACS Nano 2018, 12, 10843–10854. 10.1021/acsnano.8b03363. 30346692

[ref20] Sun B.; Pokhrel S.; Dunphy D. R.; Zhang H.; Ji Z.; Wang X.; Wang M.; Liao Y. P.; Chang C. H.; Dong J.; Li R.; Mädler L.; Brinker C. J.; Nel A. E.; Xia T. Reduction of Acute Inflammatory Effects of Fumed Silica Nanoparticles in the Lung by Adjusting Silanol Display Through Calcination and Metal Doping. ACS Nano 2015, 9, 9357–9372. 10.1021/acsnano.5b03443. 26200133 PMC4687969

[ref21] Sun B.; Wang X.; Liao Y. P.; Ji Z.; Chang C. H.; Pokhrel S.; Ku J.; Liu X.; Wang M.; Dunphy D. R.; Li R.; Meng H.; Mädler L.; Brinker C. J.; Nel A. E.; Xia T. Repetitive Dosing of Fumed Silica Leads to Profibrogenic Effects Through Unique Structure-Activity Relationships and Biopersistence in the Lung. ACS Nano 2016, 10, 8054–8066. 10.1021/acsnano.6b04143. 27483033 PMC5214959

[ref22] Lunov O.; Syrovets T.; Loos C.; Beil J.; Delacher M.; Tron K.; Nienhaus G. U.; Musyanovych A.; Mailänder V.; Landfester K.; Simmet T. Differential Uptake of Functionalized Polystyrene Nanoparticles by Human Macrophages and a Monocytic Cell Line. ACS Nano 2011, 5, 1657–1669. 10.1021/nn2000756. 21344890

[ref23] Thomen A.; Najafinobar N.; Penen F.; Kay E.; Upadhyay P. P.; Li X.; Phan N. T. N.; Malmberg P.; Klarqvist M.; Andersson S.; Kurczy M. E.; Ewing A. G. Subcellular Mass Spectrometry Imaging and Absolute Quantitative Analysis Across Organelles. ACS Nano 2020, 14, 4316–4325. 10.1021/acsnano.9b09804. 32239916 PMC7199216

[ref24] Lunov O.; Zablotskii V.; Syrovets T.; Röcker C.; Tron K.; Nienhaus G. U.; Simmet T. Modeling Receptor-Mediated Endocytosis of Polymer-Functionalized Iron Oxide Nanoparticles by Human Macrophages. Biomaterials 2011, 32, 547–555. 10.1016/j.biomaterials.2010.08.111. 20880574

[ref25] Chao Y.; Karmali P. P.; Mukthavaram R.; Kesari S.; Kouznetsova V. L.; Tsigelny I. F.; Simberg D. Direct Recognition of Superparamagnetic Nanocrystals by Macrophage Scavenger Receptor SR-AI. ACS Nano 2013, 7, 4289–4298. 10.1021/nn400769e. 23614696

[ref26] Gallud A.; Bondarenko O.; Feliu N.; Kupferschmidt N.; Atluri R.; Garcia-Bennett A.; Fadeel B. Macrophage Activation Status Determines the Internalization of Mesoporous Silica Particles: Exploring the Role of Different Pattern Recognition Receptors. Biomaterials 2017, 121, 28–40. 10.1016/j.biomaterials.2016.12.029. 28063981

[ref27] Cheng J.; Zhang Q.; Fan S.; Zhang A.; Liu B.; Hong Y.; Guo J.; Cui D.; Song J. The Vacuolization of Macrophages Induced by Large Amounts of Inorganic Nanoparticle Uptake to Enhance the Immune Response. Nanoscale 2019, 11, 22849–22859. 10.1039/C9NR08261A. 31755508

[ref28] Olzmann J. A.; Carvalho P. Dynamics and Functions of Lipid Droplets. Nat. Rev. Mol. Cell Biol. 2019, 20, 137–155. 10.1038/s41580-018-0085-z. 30523332 PMC6746329

[ref29] Zadoorian A.; Du X.; Yang H. Lipid Droplet Biogenesis and Functions in Health and Disease. Nat. Rev. Endocrinol. 2023, 19, 443 10.1038/s41574-023-00845-0. 37221402 PMC10204695

[ref30] Leibe R.; Hsiao I. L.; Fritsch-Decker S.; Kielmeier U.; Wagbo A. M.; Voss B.; Schmidt A.; Hessman S. D.; Duschl A.; Oostingh G. J.; Diabaté S.; Weiss C. The Protein Corona Suppresses the Cytotoxic and Pro-Inflammatory Response in Lung Epithelial Cells and Macrophages upon Exposure to Nanosilica. Arch. Toxicol. 2019, 93, 871–885. 10.1007/s00204-019-02422-9. 30838431

[ref31] Fritsch-Decker S.; Marquardt C.; Stoeger T.; Diabaté S.; Weiss C. Revisiting the Stress Paradigm for Silica Nanoparticles: Decoupling of the Anti-Oxidative Defense, Pro-Inflammatory Response and Cytotoxicity. Arch. Toxicol. 2018, 92, 2163–2174. 10.1007/s00204-018-2223-y. 29799070

[ref32] Yang W. S.; SriRamaratnam R.; Welsch M. E.; Shimada K.; Skouta R.; Viswanathan V. S.; Cheah J. H.; Clemons P. A.; Shamji A. F.; Clish C. B.; Brown L. M.; Girotti A. W.; Cornish V. W.; Schreiber S. L.; Stockwell B. R. Regulation of Ferroptotic Cancer Cell Death by GPX4. Cell 2014, 156, 317–331. 10.1016/j.cell.2013.12.010. 24439385 PMC4076414

[ref33] Alkhammash H. I.; Li N.; Berthier R.; de Planque M. R. Native Silica Nanoparticles are Powerful Membrane Disruptors. Phys. Chem. Chem. Phys. 2015, 17, 15547–15560. 10.1039/C4CP05882H. 25623776

[ref34] Nazemidashtarjandi S.; Vahedi A.; Farnoud A. M. Lipid Chemical Structure Modulates the Disruptive Effects of Nanomaterials on Membrane Models. Langmuir 2020, 36, 4923–4932. 10.1021/acs.langmuir.0c00295. 32312045 PMC8725912

[ref35] Mukherjee S. P.; Lazzaretto B.; Hultenby K.; Newman L.; Rodrigues A. F.; Lozano N.; Kostarelos K.; Malmberg P.; Fadeel B. Graphene Oxide Elicits Membrane Lipid Changes and Neutrophil Extracellular Trap Formation. Chem 2018, 4, 334–358. 10.1016/j.chempr.2017.12.017.

[ref36] Veith L.; Vennemann A.; Breitenstein D.; Engelhard C.; Wiemann M.; Hagenhoff B. Detection of SiO_2_ Nanoparticles in Lung Tissue by ToF-SIMS Imaging and Fluorescence Microscopy. Analyst 2017, 142, 2631–2639. 10.1039/C7AN00399D. 28608905

[ref37] Fletcher J. S.; Vickerman J. C. Secondary Ion Mass Spectrometry: Characterizing Complex Samples in Two and Three Dimensions. Anal. Chem. 2013, 85, 610–639. 10.1021/ac303088m. 23094968

[ref38] Pavan C.; Santalucia R.; Leinardi R.; Fabbiani M.; Yakoub Y.; Uwambayinema F.; Ugliengo P.; Tomatis M.; Martra G.; Turci F.; Lison D.; Fubini B. Nearly Free Surface Silanols are the Critical Molecular Moieties that Initiate the Toxicity of Silica Particles. Proc. Natl. Acad. Sci. U. S. A. 2020, 117, 27836–27846. 10.1073/pnas.2008006117. 33097669 PMC7668052

[ref39] Winter M.; Beer H. D.; Hornung V.; Krämer U.; Schins R. P.; Förster I. Activation of the Inflammasome by Amorphous Silica and TiO_2_ Nanoparticles in Murine Dendritic Cells. Nanotoxicology 2011, 5, 326–340. 10.3109/17435390.2010.506957. 20846021

[ref40] Sandberg W. J.; Låg M.; Holme J. A.; Friede B.; Gualtieri M.; Kruszewski M.; Schwarze P. E.; Skuland T.; Refsnes M. Comparison of Non-Crystalline Silica Nanoparticles in IL-1β Release from Macrophages. Part. Fibre Toxicol. 2012, 9, 32 10.1186/1743-8977-9-32. 22882971 PMC3441334

[ref41] Muñoz-Planillo R.; Kuffa P.; Martinez-Colón G.; Smith B. L.; Rajendiran T. M.; Nuñez G. K^+^ Efflux is the Common Trigger of NLRP3 Inflammasome Activation by Bacterial Toxins and Particulate Matter. Immunity 2013, 38, 1142–1153. 10.1016/j.immuni.2013.05.016. 23809161 PMC3730833

[ref42] Riteau N.; Baron L.; Villeret B.; Guillou N.; Savigny F.; Ryffel B.; Rassendren F.; Le Bert M.; Gombault A.; Couillin I. ATP Release and Purinergic Signaling: a Common Pathway for Particle-Mediated Inflammasome Activation. Cell Death Dis. 2012, 3, e403 10.1038/cddis.2012.144. 23059822 PMC3481132

[ref43] Baron L.; Gombault A.; Fanny M.; Villeret B.; Savigny F.; Guillou N.; Panek C.; Le Bert M.; Lagente V.; Rassendren F.; Riteau N.; Couillin I. The NLRP3 Inflammasome is Activated by Nanoparticles Through ATP, ADP and Adenosine. Cell Death Dis. 2015, 6, e1629 10.1038/cddis.2014.576. 25654762 PMC4669808

[ref44] Andón F. T.; Mukherjee S. P.; Gessner I.; Wortmann L.; Xiao L.; Hultenby K.; Shvedova A. A.; Mathur S.; Fadeel B. Hollow Carbon Spheres Trigger Inflammasome-Dependent IL-1β Secretion in Macrophages. Carbon 2017, 113, 243–251. 10.1016/j.carbon.2016.11.049.

[ref45] Bhattacharya K.; Kiliç G.; Costa P. M.; Fadeel B. Cytotoxicity Screening and Cytokine Profiling of Nineteen Nanomaterials Enables Hazard Ranking and Grouping Based on Inflammogenic Potential. Nanotoxicology 2017, 11, 809–826. 28816564 10.1080/17435390.2017.1363309

[ref46] Gaidt M. M.; Ebert T. S.; Chauhan D.; Schmidt T.; Schmid-Burgk J. L.; Rapino F.; Robertson A. A.; Cooper M. A.; Graf T.; Hornung V. Human Monocytes Engage an Alternative Inflammasome Pathway. Immunity 2016, 44, 833–846. 10.1016/j.immuni.2016.01.012. 27037191

[ref47] Keshavan S.; Gupta G.; Martin S.; Fadeel B. Multi-Walled Carbon Nanotubes Trigger Lysosome-Dependent Cell Death (Pyroptosis) in Macrophages but not in Neutrophils. Nanotoxicology 2021, 15, 1125–1150. 10.1080/17435390.2021.1988171. 34657549

[ref48] Palomäki J.; Välimäki E.; Sund J.; Vippola M.; Clausen P. A.; Jensen K. A.; Savolainen K.; Matikainen S.; Alenius H. Long, Needle-Like Carbon Nanotubes and Asbestos Activate the NLRP3 Inflammasome Through a Similar Mechanism. ACS Nano 2011, 5, 6861–6870. 10.1021/nn200595c. 21800904

[ref49] Vakurov A.; Brydson R.; Nelson A. Electrochemical Modeling of the Silica Nanoparticle-Biomembrane Interaction. Langmuir. 2012, 28, 1246–1255. 10.1021/la203568n. 22142270

[ref50] Vakurov A.; Drummond-Brydson R.; William N.; Sanver D.; Bastus N.; Moriones O. H.; Puntes V.; Nelson A. L. Heterogeneous Rate Constant for Amorphous Silica Nanoparticle Adsorption on Phospholipid Monolayers. Langmuir 2022, 38, 5372–5380. 10.1021/acs.langmuir.1c03155. 35471829 PMC9097521

[ref51] Lozano O.; Silva-Platas C.; Chapoy-Villanueva H.; Pérez B. E.; Lees J. G.; Ramachandra C. J. A.; Contreras-Torres F. F.; Lázaro-Alfaro A.; Luna-Figueroa E.; Bernal-Ramírez J.; Gordillo-Galeano A.; Benitez A.; Oropeza-Almazán Y.; Castillo E. C.; Koh P. L.; Hausenloy D. J.; Lim S. Y.; García-Rivas G. Amorphous SiO_2_ Nanoparticles Promote Cardiac Dysfunction *via* the Opening of the Mitochondrial Permeability Transition Pore in Rat Heart and Human Cardiomyocytes. Part. Fibre Toxicol. 2020, 17, 15 10.1186/s12989-020-00346-2. 32381100 PMC7206702

[ref52] Inoue M.; Sakamoto K.; Suzuki A.; Nakai S.; Ando A.; Shiraki Y.; Nakahara Y.; Omura M.; Enomoto A.; Nakase I.; Sawada M.; Hashimoto N. Size and Surface Modification of Silica Nanoparticles Affect the Severity of Lung Toxicity by Modulating Endosomal ROS Generation in Macrophages. Part. Fibre Toxicol. 2021, 18, 21 10.1186/s12989-021-00415-0. 34134732 PMC8210371

[ref53] de Planque M. R.; Aghdaei S.; Roose T.; Morgan H. Electrophysiological Characterization of Membrane Disruption by Nanoparticles. ACS Nano 2011, 5, 3599–3606. 10.1021/nn103320j. 21517083

[ref54] Wang B.; Tontonoz P. Phospholipid Remodeling in Physiology and Disease. Annu. Rev. Physiol. 2019, 81, 165–188. 10.1146/annurev-physiol-020518-114444. 30379616 PMC7008953

[ref55] Balsinde J.; Bianco I. D.; Ackermann E. J.; Conde-Frieboes K.; Dennis E. A. Inhibition of Calcium-Independent Phospholipase A_2_ Prevents Arachidonic Acid Incorporation and Phospholipid Remodeling in P388D1 Macrophages. Proc. Natl. Acad. Sci. U. S. A. 1995, 92, 8527–8531. 10.1073/pnas.92.18.8527. 7667324 PMC41190

[ref56] Balsinde J.; Balboa M. A.; Dennis E. A. Antisense Inhibition of Group VI Ca^2+^-Independent Phospholipase A_2_ Blocks Phospholipid Fatty Acid Remodeling in Murine P388D1Macrophages. J. Biol. Chem. 1997, 272, 29317–29321. 10.1074/jbc.272.46.29317. 9361012

[ref57] Sun W. Y.; Tyurin V. A.; Mikulska-Ruminska K.; Shrivastava I. H.; Anthonymuthu T. S.; Zhai Y. J.; Pan M. H.; Gong H. B.; Lu D. H.; Sun J.; Duan W. J.; Korolev S.; Abramov A. Y.; Angelova P. R.; Miller I.; Beharier O.; Mao G. W.; Dar H. H.; Kapralov A. A.; Amoscato A. A.; Hastings T. G.; Greenamyre T. J.; Chu C. T.; Sadovsky Y.; Bahar I.; Bayir H.; Tyurina Y. Y.; He R. R.; Kagan V. E. Phospholipase iPLA_2_β Averts Ferroptosis by Eliminating a Redox Lipid Death Signal. Nat. Chem. Biol. 2021, 17, 465–476. 10.1038/s41589-020-00734-x. 33542532 PMC8152680

[ref58] Chen D.; Chu B.; Yang X.; Liu Z.; Jin Y.; Kon N.; Rabadan R.; Jiang X.; Stockwell B. R.; Gu W. iPLA2β-Mediated Lipid Detoxification Controls p53-Driven Ferroptosis Independent of GPX4. Nat. Commun. 2021, 12, 3644 10.1038/s41467-021-23902-6. 34131139 PMC8206155

[ref59] Balsinde J.; Dennis E. A. Bromoenol Lactone Inhibits Magnesium-Dependent Phosphatidate Phosphohydrolase and Blocks Triacylglycerol Biosynthesis in Mouse P388D1Macrophages. J. Biol. Chem. 1996, 271, 31937–31941. 10.1074/jbc.271.50.31937. 8943239

[ref60] Liu-Wu Y.; Hurt-Camejo E.; Wiklund O. Lysophosphatidylcholine Induces the Production of IL-1β by Human Monocytes. Atherosclerosis 1998, 137, 351–357. 10.1016/S0021-9150(97)00295-5. 9622278

[ref61] Magupalli V. G.; Negro R.; Tian Y.; Hauenstein A. V.; Di Caprio G.; Skillern W.; Deng Q.; Orning P.; Alam H. B.; Maliga Z.; Sharif H.; Hu J. J.; Evavold C. L.; Kagan J. C.; Schmidt F. I.; Fitzgerald K. A.; Kirchhausen T.; Li Y.; Wu H. HDAC6 Mediates an Aggresome-like Mechanism for NLRP3 and Pyrin Inflammasome Activation. Science 2020, 369, eaas8995 10.1126/science.aas8995. 32943500 PMC7814939

[ref62] Kang R.; Zeng L.; Zhu S.; Xie Y.; Liu J.; Wen Q.; Cao L.; Xie M.; Ran Q.; Kroemer G.; Wang H.; Billiar T. R.; Jiang J.; Tang D. Lipid Peroxidation Drives Gasdermin D-Mediated Pyroptosis in Lethal Polymicrobial Sepsis. Cell Host Microbe 2018, 24, 97–108.e4. 10.1016/j.chom.2018.05.009. 29937272 PMC6043361

[ref63] Lehman S. E.; Morris A. S.; Mueller P. S.; Salem A. K.; Grassian V. H.; Larsen S. C. Silica Nanoparticle-Generated ROS as a Predictor of Cellular Toxicity: Mechanistic Insights and Safety by Design. Environ. Sci.: Nano 2016, 3, 56–66. 10.1039/C5EN00179J. 26998307 PMC4795909

[ref64] Gupta G.; Gliga A.; Hedberg J.; Serra A.; Greco D.; Odnevall Wallinder I.; Fadeel B. Cobalt Nanoparticles Trigger Ferroptosis-Like Cell Death (Oxytosis) in Neuronal Cells: Potential Implications for Neurodegenerative Disease. FASEB J. 2020, 34, 5262–5281. 10.1096/fj.201902191RR. 32060981

[ref65] Vanbellingen Q. P.; Elie N.; Eller M. J.; Della-Negra S.; Touboul D.; Brunelle A. Time-of-Flight Secondary Ion Mass Spectrometry Imaging of Biological Samples with Delayed Extraction for High Mass and High Spatial Resolutions. Rapid Commun. Mass Spectrom. 2015, 29, 1187–1195. 10.1002/rcm.7210. 26395603 PMC5033000

