# Supplementary material for: Exploiting Mass Spectrometry to Unlock the Mechanism of Nanoparticle-Induced Inflammasome Activation
Source: ACS Nano. 2023 Aug 29;17(17):17451–67. doi: 10.1021/acsnano.3c05600 (PMC10510732; doi:10.1021/acsnano.3c05600)
Supplement: Supplementary file 1 — nn3c05600_si_001.pdf [file nn3c05600_si_001.pdf]

SUPPORTING INFORMATION

Exploiting Mass Spectrometry to Unlock the Mechanism of Nanoparticle-Induced Inflammasome Activation

Govind Gupta[1†], Jasreen Kaur[1], Kunal Bhattacharya[1‡], Benedict J. Chambers[2], Arianna Gazzi[3], Giulia Furesi[4], Martina Rauner[4], Claudia Fuoco[5], Marco Orecchioni[6], Lucia Gemma Delogu[3], Lars Haag[7], Jan Eric Stehr[8], Aurélien Thomen[9], Romain Bordes[10], Per Malmberg[10], Gulaim A. Seisenbaeva[11], Vadim G. Kessler[11], Michael Persson[10] and Bengt Fadeel[1*]

[1]Institute of Environmental Medicine, Karolinska Institutet, 171 77 Stockholm, Sweden; [2]Department of Medicine Huddinge, Karolinska Institutet, 141 52 Huddinge, Sweden; [3]Department of Biomedical Sciences, University of Padua, Padua 35121, Italy; [4]Department of Medicine III, and Center for Healthy Aging, TU Dresden, 01307 Dresden, Germany; [5]Department of Biology, University of Rome Tor Vergata, Rome 00173, Italy; [6]Division of Inflammation Biology, La Jolla Institute for Immunology, La Jolla, CA 92037, USA; [7]Department of Laboratory Medicine, Karolinska Institutet, 141 52 Huddinge, Sweden; [8]Department of Physics, Chemistry and Biology, Linköping University, 581 83 Linköping, Sweden; [9]Department of Chemistry and Molecular Biology, University of Gothenburg, 412 96 Göteborg, Sweden; [10]Department of Chemistry and Chemical Engineering, Chalmers University of Technology, 412 96 Göteborg, Sweden; [11]Department of Molecular Sciences, Swedish University of Agricultural Sciences, 750 07 Uppsala, Sweden.

[†]Present affiliation: Swiss Federal Laboratories for Materials Science and Technology (EMPA), 9014 St. Gallen, Switzerland.

[‡]Present affiliation: Drug Discovery and Development Division, Patanjali Research Institute, Haridwar, Uttarakhand, 249405, India.

*Correspondence: Division of Molecular Toxicology, Institute of Environmental Medicine, Nobels väg 13, Karolinska Institutet, 171 77 Stockholm, Sweden; E-mail: bengt.fadeel@ki.se

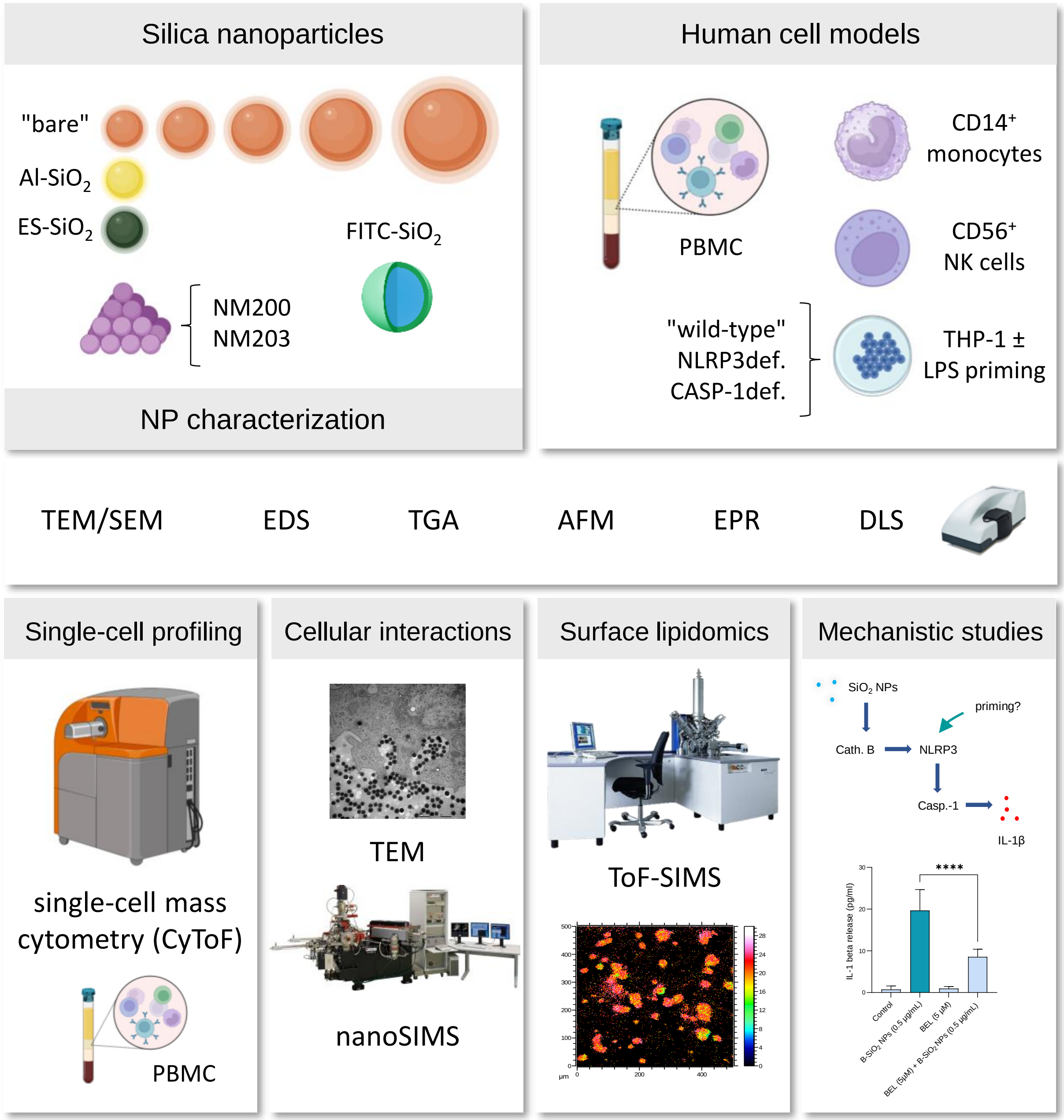

Scheme S1: The study design is schematically depicted. In the present study, a panel of amorphous silica nanoparticles (NPs) of varying sizes and with different surface properties were evaluated with respect to cell death and cytokine secretion using primary human cells, i.e., peripheral blood mononuclear cells (PBMCs) and purified CD14+ monocytes or purified CD56+ natural killer (NK) cells, as well as cell lines, i.e., THP-1 cells with monocyte-like features (non-differentiated) or macrophage-like properties (PMA-differentiated). Additionally, THP-1 cells with stable knockdown of NLRP3 or caspase-1 were applied to explore the role of the so-called inflammasome. For some experiments, the cells were primed with LPS, but for the most part, the present studies were conducted in the absence of priming with a microbial ligand. To evaluate the impact of silica NPs on cells, we deployed several mass spectrometry-based techniques: single-cell mass cytometry or cytometry by time-of-flight (CyToF), nanoscale secondary ion mass spectrometry (nanoSIMS), and time-of-flight secondary ion mass spectrometry (ToF-SIMS). Some elements of the figure were prepared with BioRender.com under an academic license.

Table S1: Physicochemical characterization of a panel of amorphous silica nanoparticles.

	SiO_2 NPs[a]	Size (nm)[b] (ES-DMA)	Size (nm)[c] (TEM)	Hydrodynamic size (nm)[d]		Zeta potential (mV)	
				MilliQ water	Cell medium	MilliQ water	Cell medium
1	CS1 (B)	17	11.5 ± 1.5	10 ± 0.49	21 ± 0.25	-42 ± 3	-19 ± 3
2	CS2 (Al)	20	N.A.	16 ± 0.08	27 ± 0.1	-31 ± 4	-16 ± 2
3	CS3 (ES)	18	N.A.	10 ± 0.04	25 ± 0.18	-12 ± 1	-4 ± 1
4	CS4	21	15.5 ± 3.4	12 ± 0.12	26 ± 0.1	-31 ± 4	-23 ± 3
5	CS5	30	23.9 ± 5.4	19 ± 0.16	52 ± 0.48	-26 ± 2	-16 ± 2
6	CS6	66	74.4 ± 17.0	89 ± 41.6	3216 ± 561	-49 ± 3	-11 ± 1
7	CS7	88	97.6 ± 12.9	98 ± 1.39	3031 ± 400	-38 ± 3	-15 ± 3
8	CS-FITC	N.A.	40	87 ± 0.3	297 ± 17	-47 ± 0.5	-11 ± 1
9	NM200	N.A.	14 ± 7	226 ± 5.0	210 ± 8.2	-22 ± 0.3	-8 ± 0.4
10	NM203	N.A.	13 ± 6	140 ± 1.2	237 ± 0.9	-30 ± 1	-8 ± 2

[a] Colloidal silica (CS) NPs 1-7 provided by Nouryon PPC (Bohus) (B, bare; Al, aluminized; ES, silane modifed); CS-FITC NPs were synthesized as described in Methods; NM200 and NM203 were obtained from the nanomaterial repository at the Joint Research Centre (JRC) of the European Commission [Rasmussen K., et al. Synthetic Amorphous Silicon Dioxide (NM-200, NM-201, NM-202, NM-203, NM-204): Characterisation and Physico-Chemical Properties. EUR 26046. Luxembourg: Publications Office of the European Union; 2013. JRC83506. EUR 26046].

[b] Electrospray-differential mobility analysis (ES-DMA) results were obtained from: Book F., et al. Ecotoxicity screening of seven different types of commercial silica nanoparticles using cellular and organismic assays: importance of surface and size. *NanoImpact*. 2019;13:100-111.

[c] TEM images shown in Figure S1-S2 were used for size distribution analysis of samples CS1-7; at least 50 NPs scored per sample. For NM200, NM203, refer to: Rasmussen K., et al. (2013).

[d] RPMI-1640 supplemented with 10% FBS, L-glutamine (2 mM), penicillin & streptomycin (100 µg/mL). PBMCs, monocytes, NK cells, and the THP-1 cell line were cultured in the same medium.

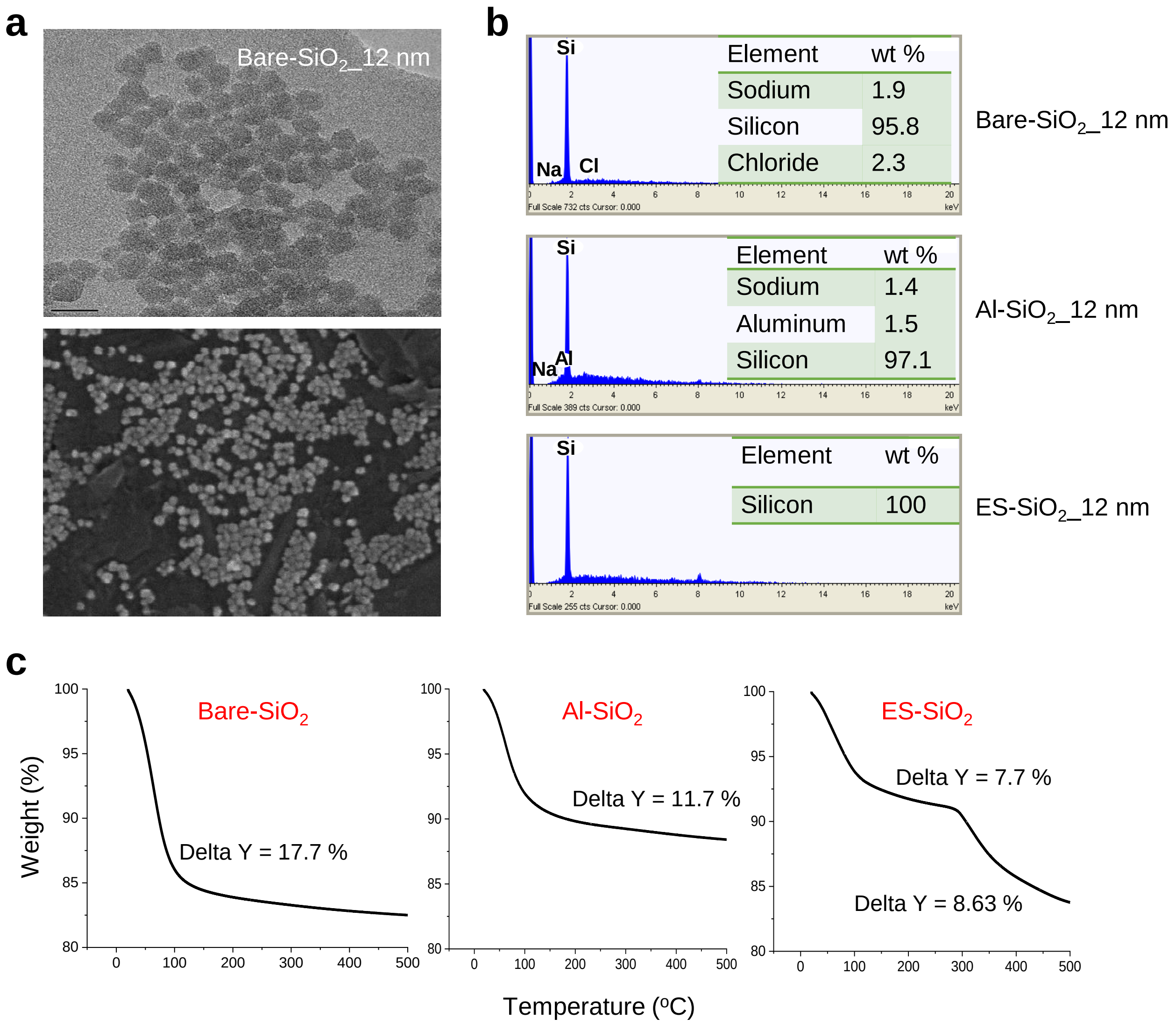

Figure S1: Characterization of silica NPs. (a) TEM and SEM images of small (12 nm) bare SiO_2 NPs. (b) EDS and (c) TGA results for bare, Al-doped, and silane-modified SiO_2 NPs.

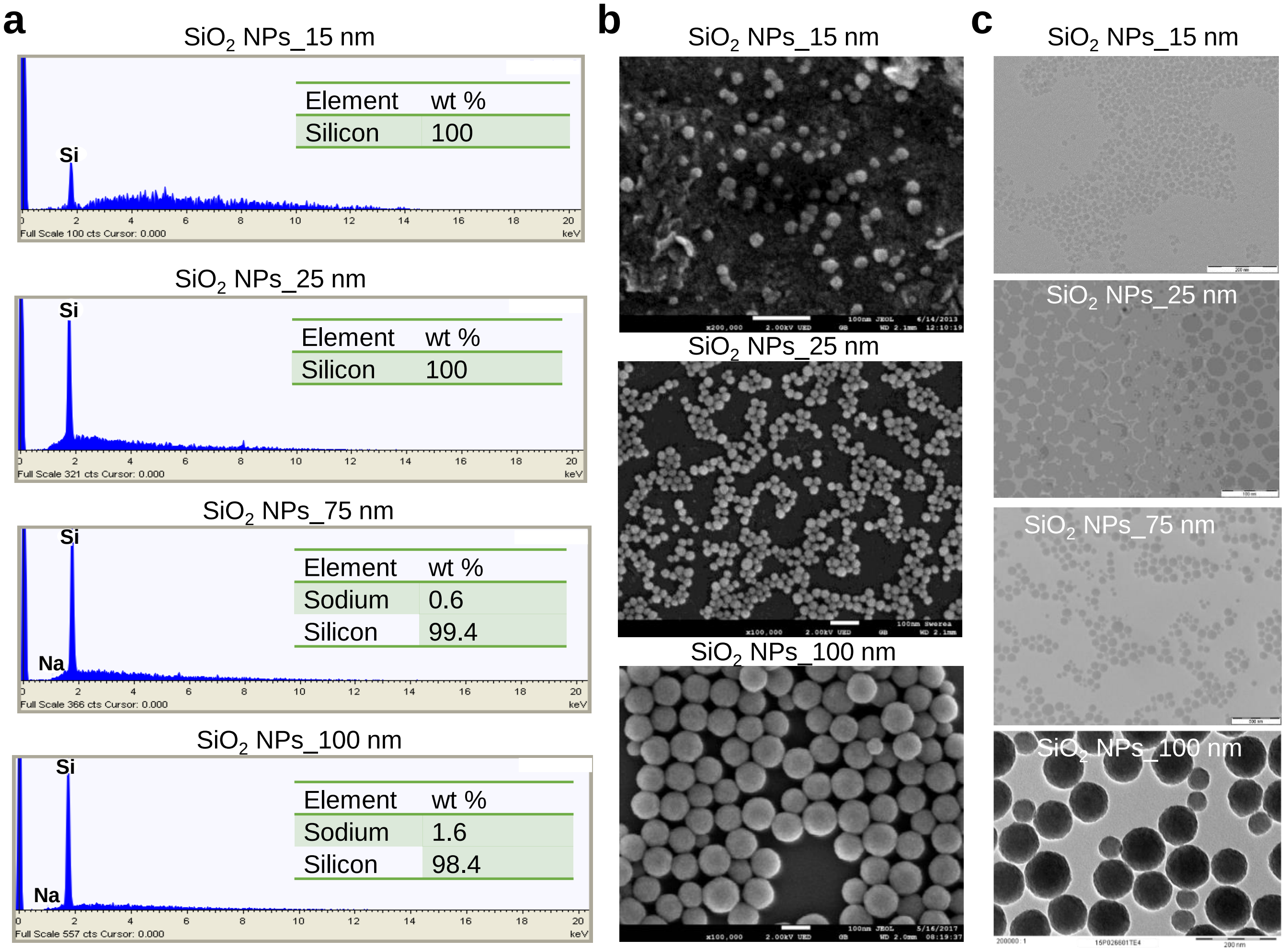

Figure S2: Characterization of silica NPs. (a) EDS results for bare amorphous SiO_2 NPs of varying sizes. (b,c) SEM and TEM images of SiO_2 NPs of varying sizes (15 – 100 nm). Refer to Table S1 for details.

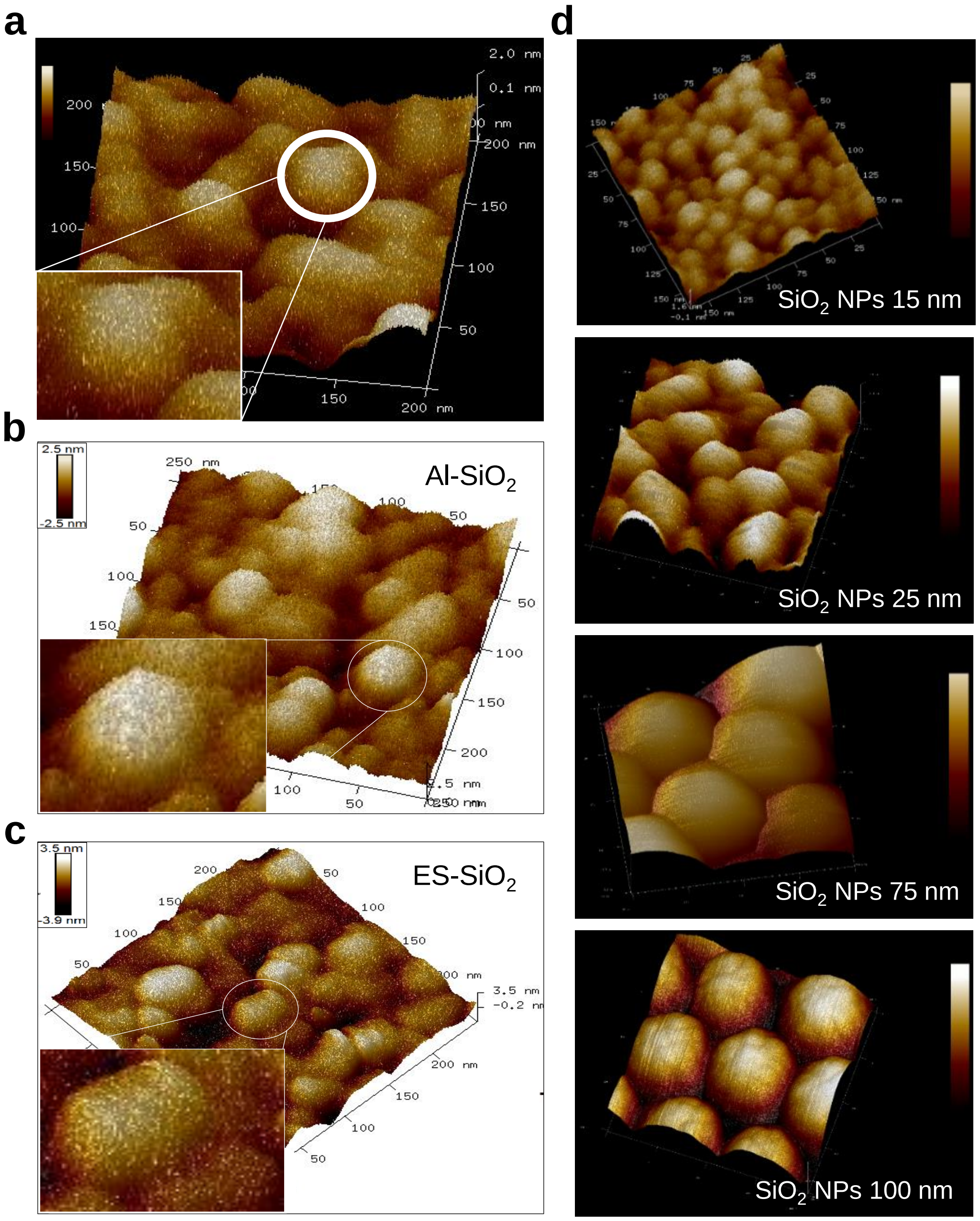

Figure S3: Physicochemical characterization of amorphous silica NPs. (a-c) AFM analysis of small (12 nm) uncoated/bare, Al-doped, and silane modified silica NPs, respectively. (d) AFM analysis of bare silica NPs ranging in size from 15 nm to 100 nm.

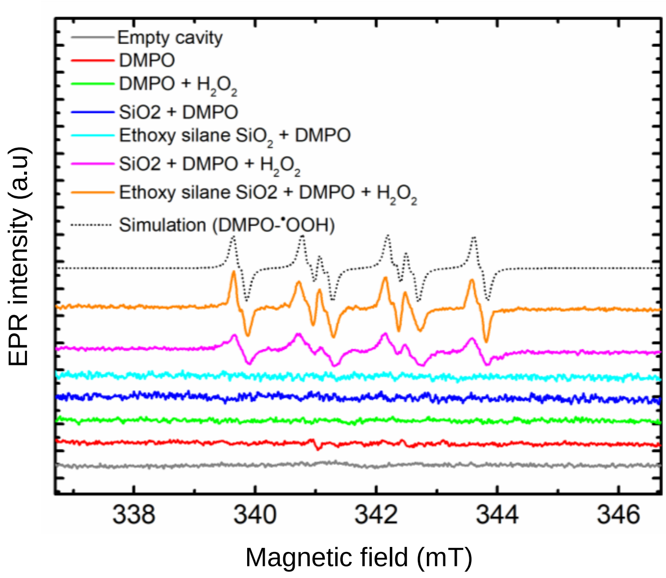

Figure S4: EPR spectra show hydroxyl radical production by bare and ethoxy silane (ES)-modified SiO_2 NPs in the presence of H_2O_2 (200 mM) and the DMPO spin trap (25 mM).

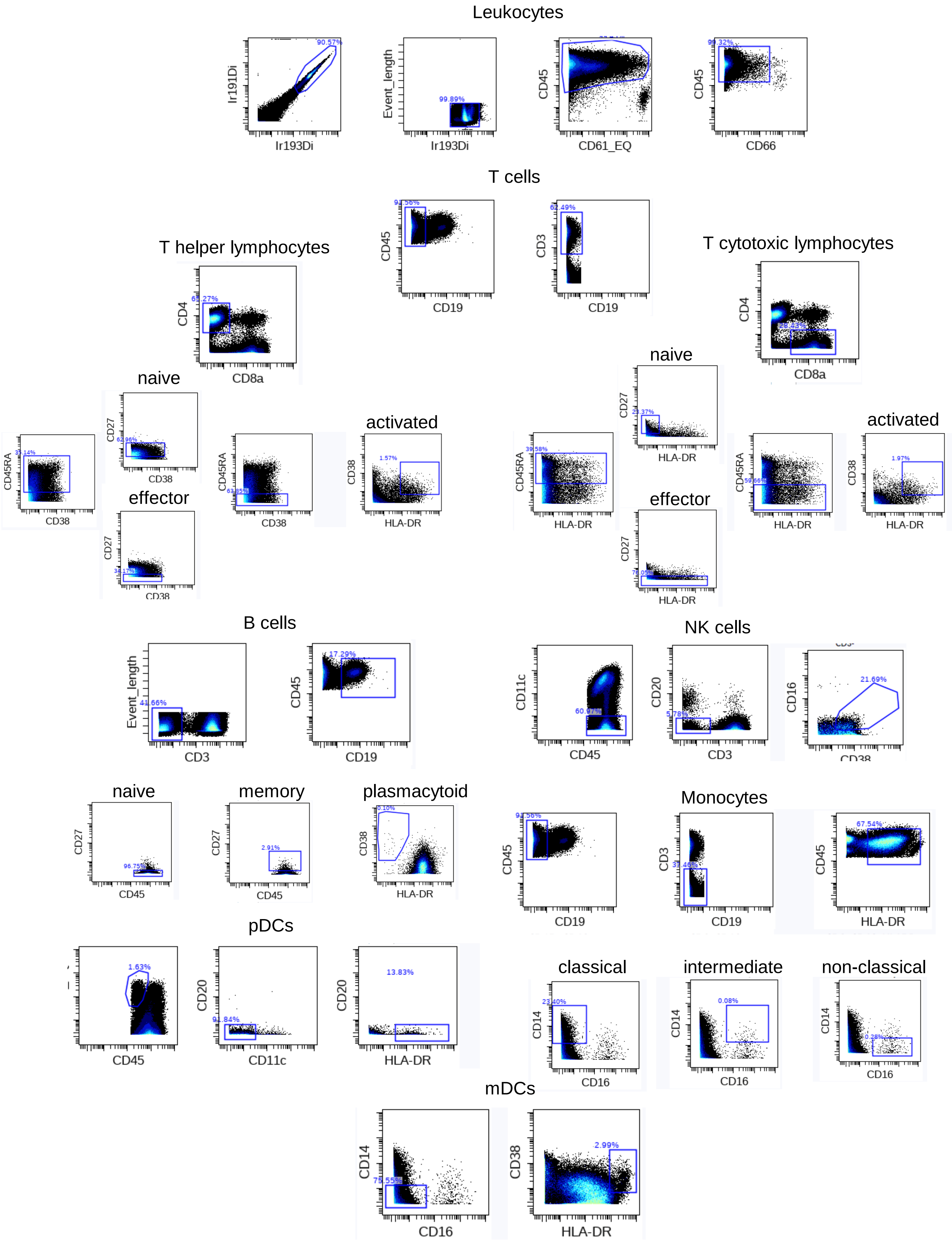

Figure S5: Gating strategy for immune cell subpopulations. FACS plots show the gating strategy used for the identification of the different immune cell subpopulations analyzed by CyTOF (refer to Figure 1).

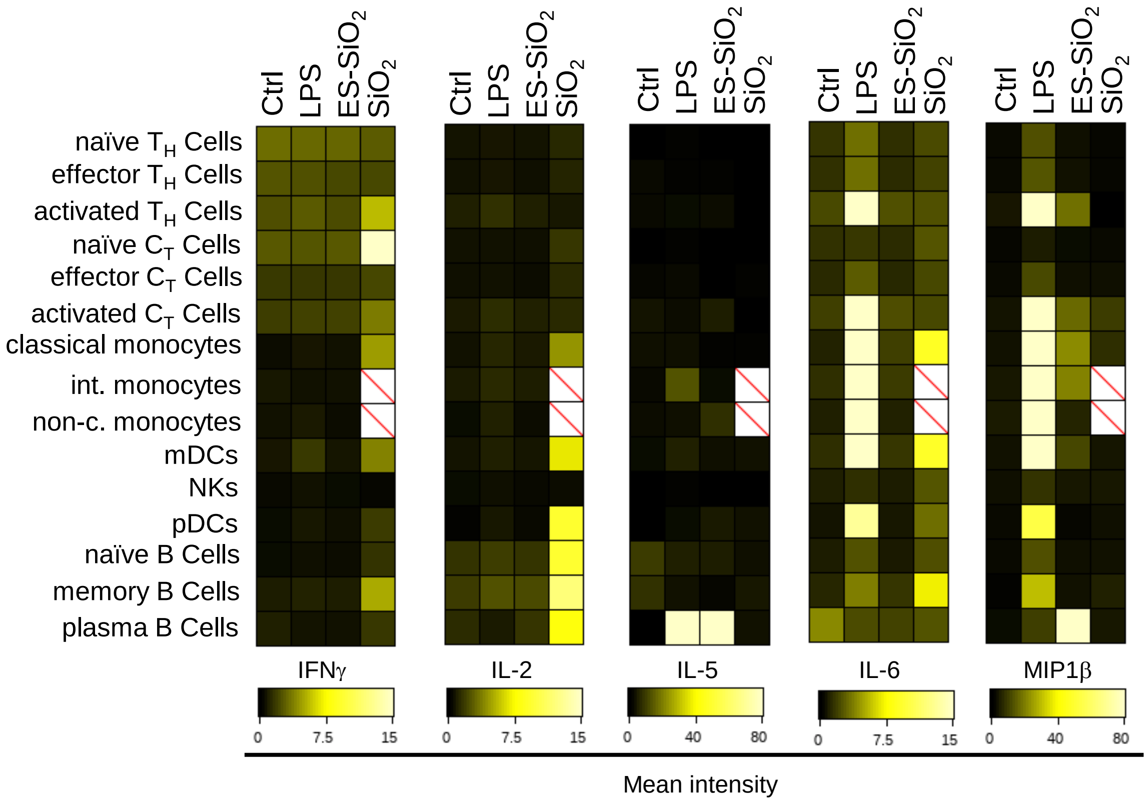

Figure S6: Single-cell analysis of cytokine production in bare SiO_2 and ES-SiO_2 exposed PBMCs (0.1 µg/mL for 24 h). LPS-treated cells were used as a positive control. Heat maps show mean marker expression ratios for gated immune cell populations for the indicated cytokines.

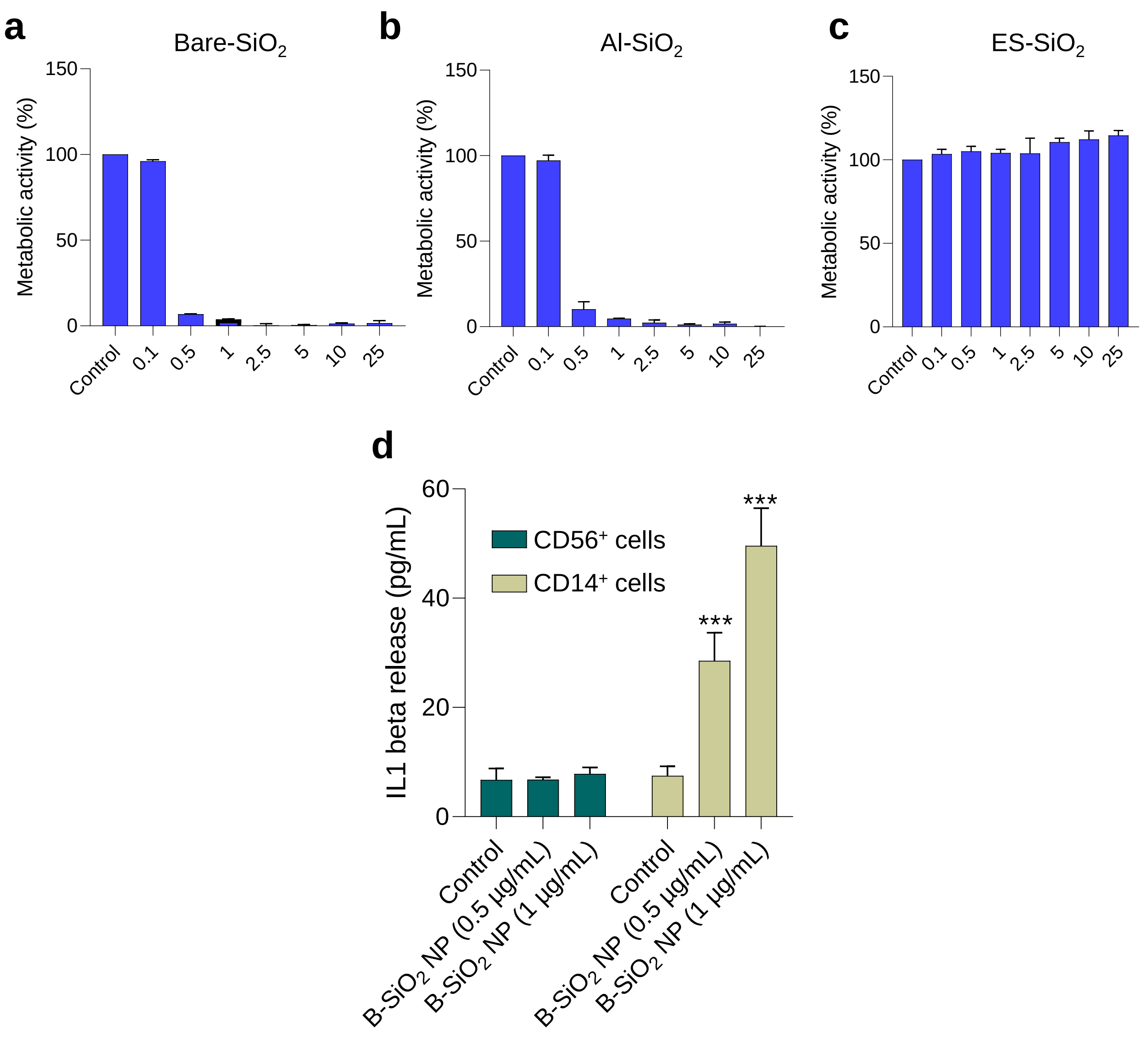

Figure S7: Cell viability and cytokine secretion in natural killer (NK) cells. (a-c) Cell viability of primary human NK cells as determined by Alamar blue assay following exposure for 24 h to bare, Al-doped, and silane modified silica NPs. Data shown are mean values ± S.D. using cells isolated from three independent donors. Corresponding results for primary human monocytes are shown in Figure 1a. (d) IL-1β secretion in primary human NK cells ($CD56^+$) *versus* monocytes ($CD14^+$) exposed for 6 h to bare silica NPs. Note that the cells were not LPS-primed. Data shown as mean values ± S.D. (n=3). ***$p<0.001$.

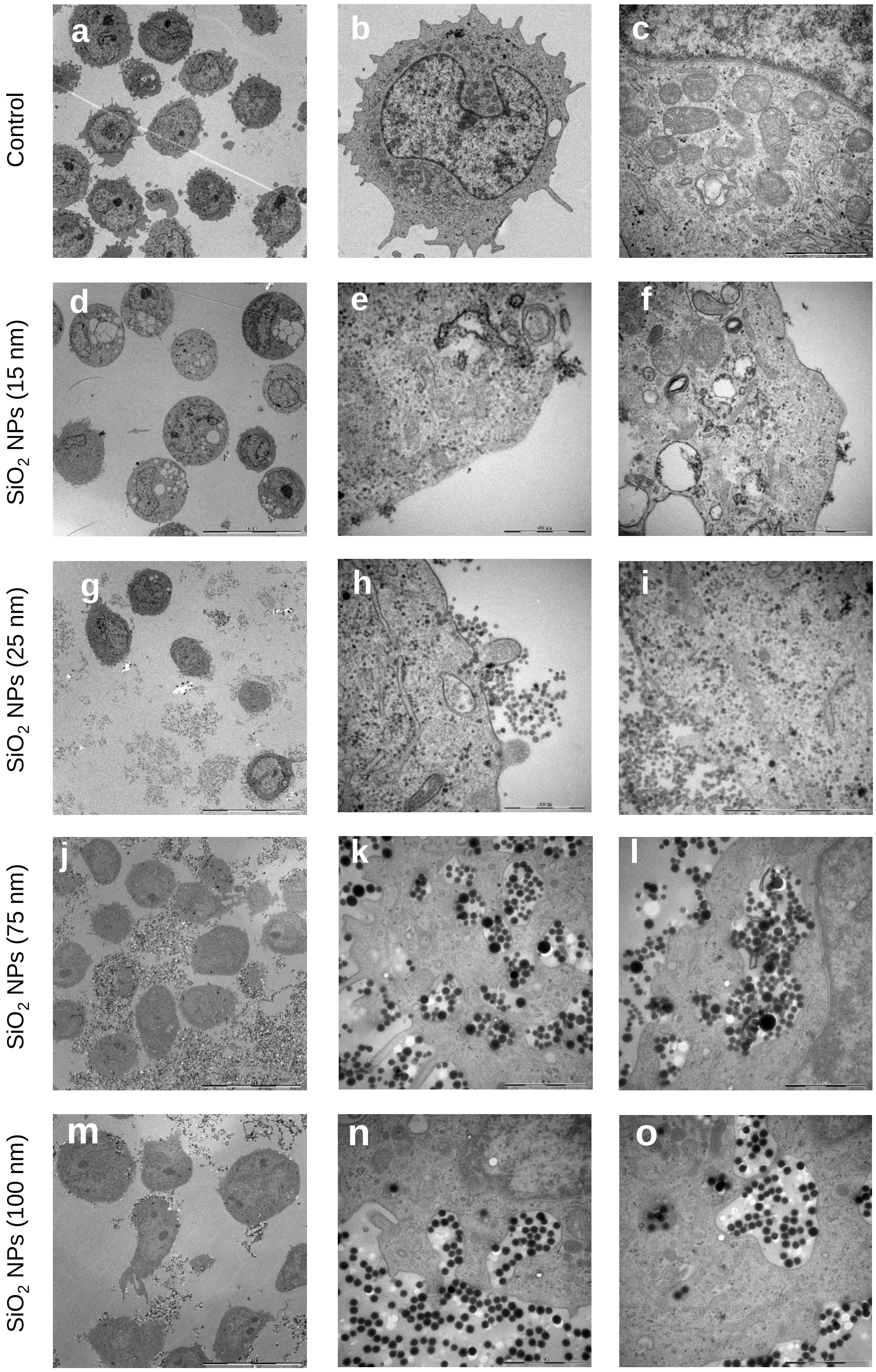

Figure S8: TEM images of THP-1 cells show cellular uptake of SiO_2 NPs of different particle sizes after 2 h of exposure at 2.5 µg/mL. (a – c) control, (d – f) 15 nm SiO_2 NPs, (g – i) 25 nm SiO_2 NPs, (j – l) 75 nm SiO_2 NPs, (m – o) 100 nm SiO_2 NPs. Refer to Figure 2a for bare and ES-modified 12 nm NPs.

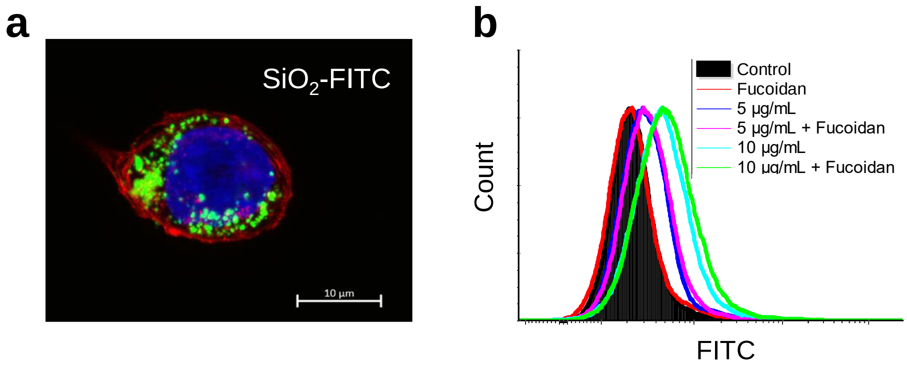

Figure S9: Cellular uptake of FITC-labelled SiO_2 NPs. (a) Confocal microscopy shows the cellular uptake of FITC-labelled (green) SiO_2 NPs (50 µg/mL) after exposure of THP-1 cells for 6 h; cells were co-stained with phalloidin-red, and counterstained with DAPI (blue) to visualize cell nuclei. (b) Uptake of FITC-labeled SiO_2 NPs (5 or 10 µg/mL) following 6 h of exposure as determined by flow cytometry in the presence and absence of the scavenger receptor inhibitor fucoidan.

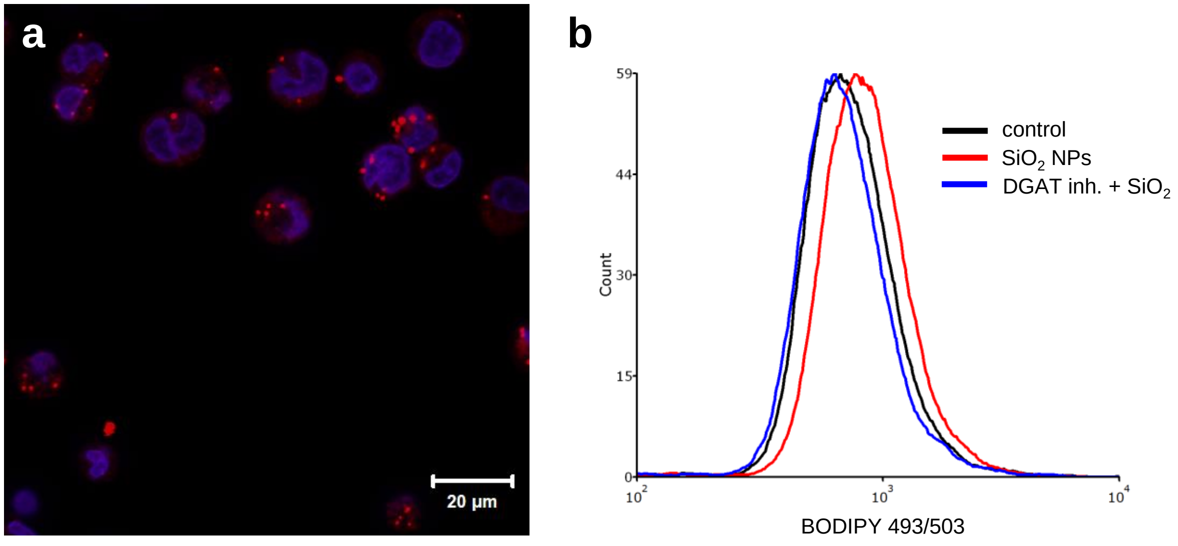

Figure S10: Silica NPs promote lipid droplet formation. (a) Lipid droplets were visualized by staining the cells with Nile Red. Cells were counterstained with DAPI. (b) Lipid droplet content was quantified using flow cytometry after labelling the cells with BODIPY 493/503. The cells were exposed to silica NPs in the presence or absence of the DGAT1 inhibitor, A 922500 (5 µM). Experiments were also conducted using the DGAT2 inhibitor, PF-06424439, but no effect was noted on silica-induced lipid droplet content (data not shown).

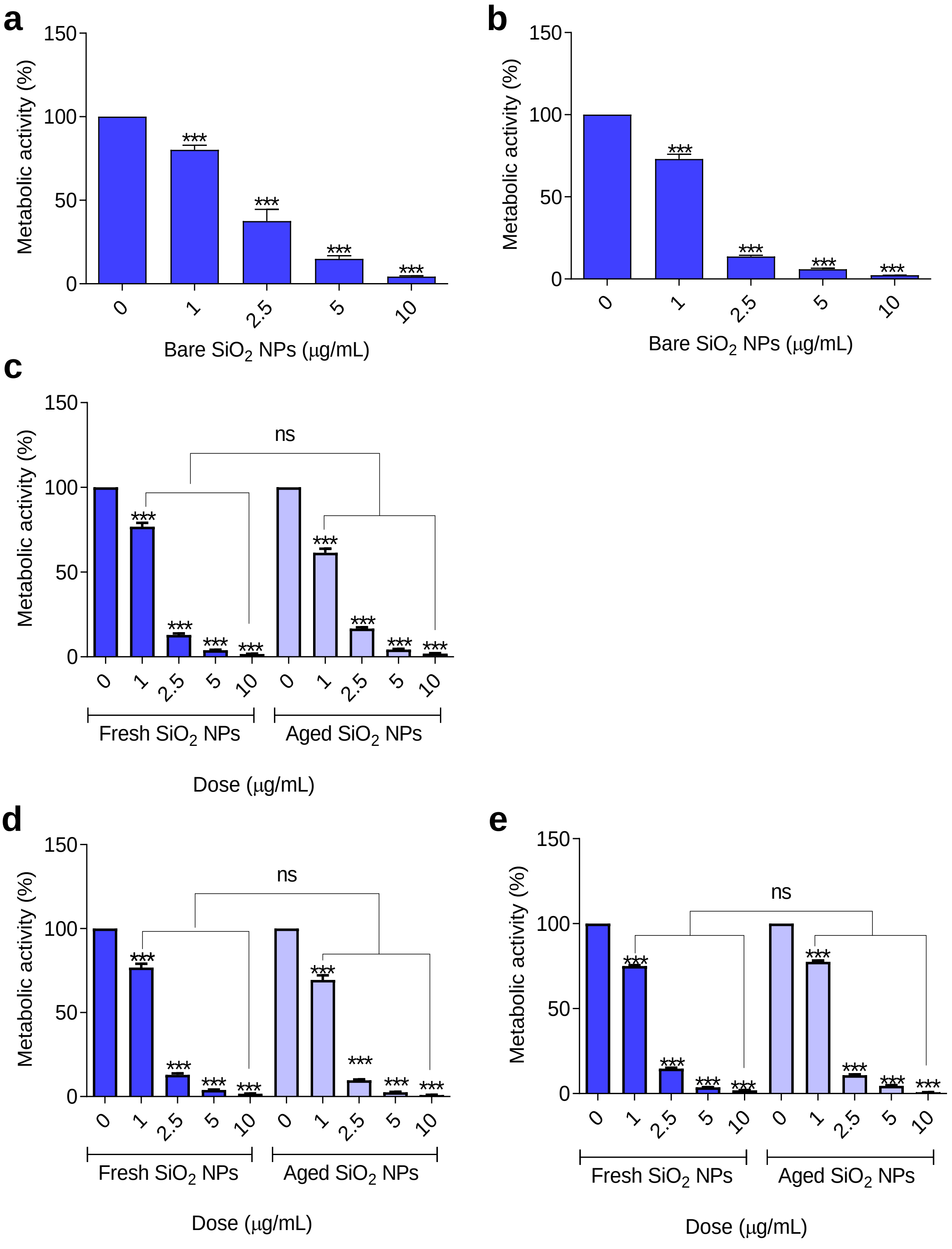

Figure S11: Toxicity of ion-exchanged and aged (7 day) small, bare SiO_2 NPs in THP-1 cells. (a-b) Metabolic activity of cells after 12 and 24 h of exposure to ion-exchanged SiO_2 NPs. (c-e) Toxicity of fresh *versus* 7-day aged silica NPs in different media: (c) medium with 10% serum, (d) medium without serum, and (e) silica NPs aged in MilliQ water and added to cells in complete medium for 24 h.

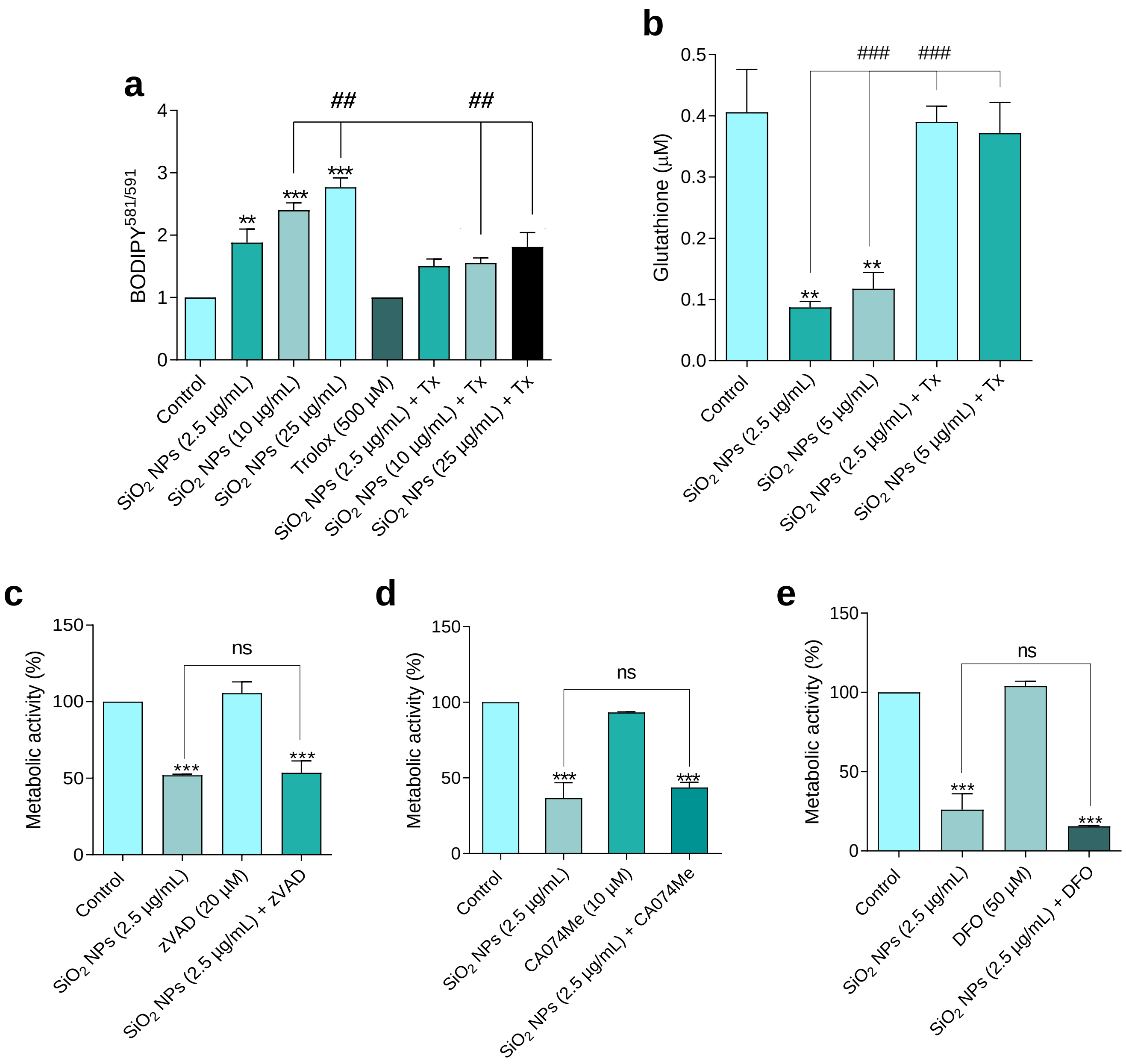

Figure S12: SiO_2 NPs trigger lipid peroxidation-dependent cell death in the monocyte-like THP-1 cell line. (a) Lipid peroxidation was determined by C11-BODIPY$^{581/591}$ labelling of cells exposed to bare SiO_2 NPs for 6 h with and without co-exposure to Trolox, a lipid antioxidant. Fluorescence was quantified by flow cytometry. (b) GSH levels were reduced after 6 h of exposure to bare SiO_2 NPs as shown using the GSH-Glow™ assay. Trolox prevented the loss of GSH. (c-e) Exploring the mechanism of cell death triggered by SiO_2 NPs. THP-1 cells were exposed to bare SiO_2 NPs for 12 h in the presence or absence of the indicated inhibitors. The pan-caspase inhibitor, zVAD-fmk (c), cathepsin B inhibitor, Ca074Me (d), and iron-chelating agent, DFO (e) all failed to prevent cell death. Data shown as mean values ± S.D. (n=3). **p<0.01, ***p<0.001, ##p< 0.01, ###p< 0.001.

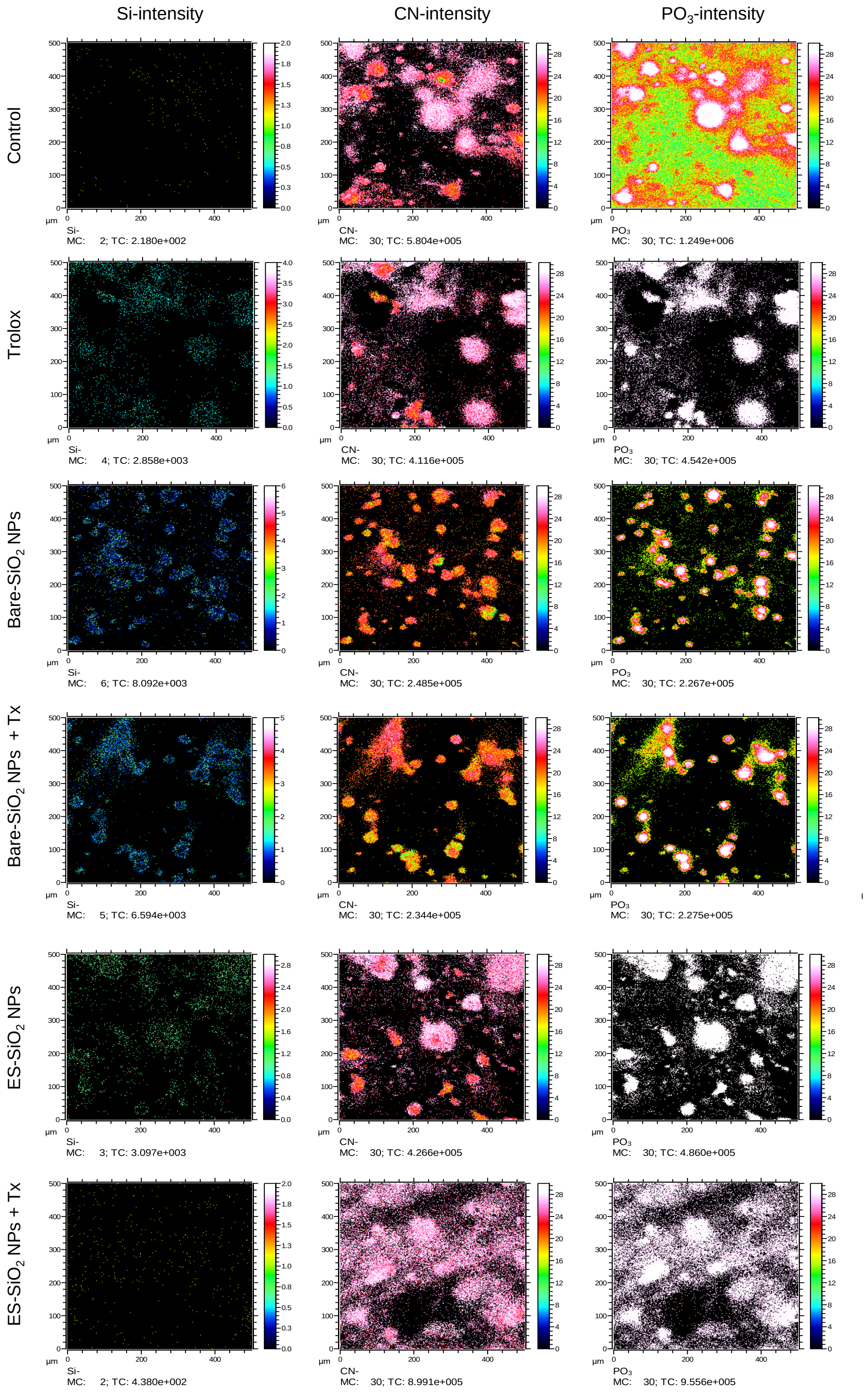

Figure S13: ToF-SIMS imaging indicates changes in overall nitrogen (CN^-) and phosphate (PO_3) as well as silica (Si) intensity in THP-1 cells after 6 h of exposure to 2.5 µg/mL small (12 nm) bare *versus* silane modified silica NPs ± Trolox.

a

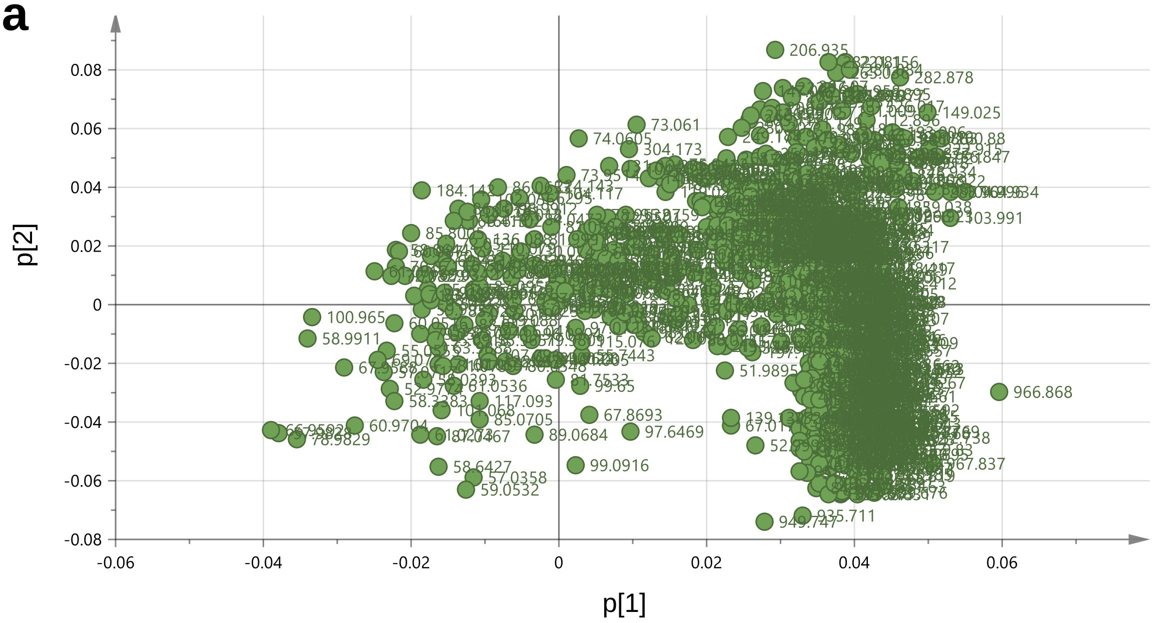

R2x[1] = 0.42 ; R2x[2] = 0.275

b

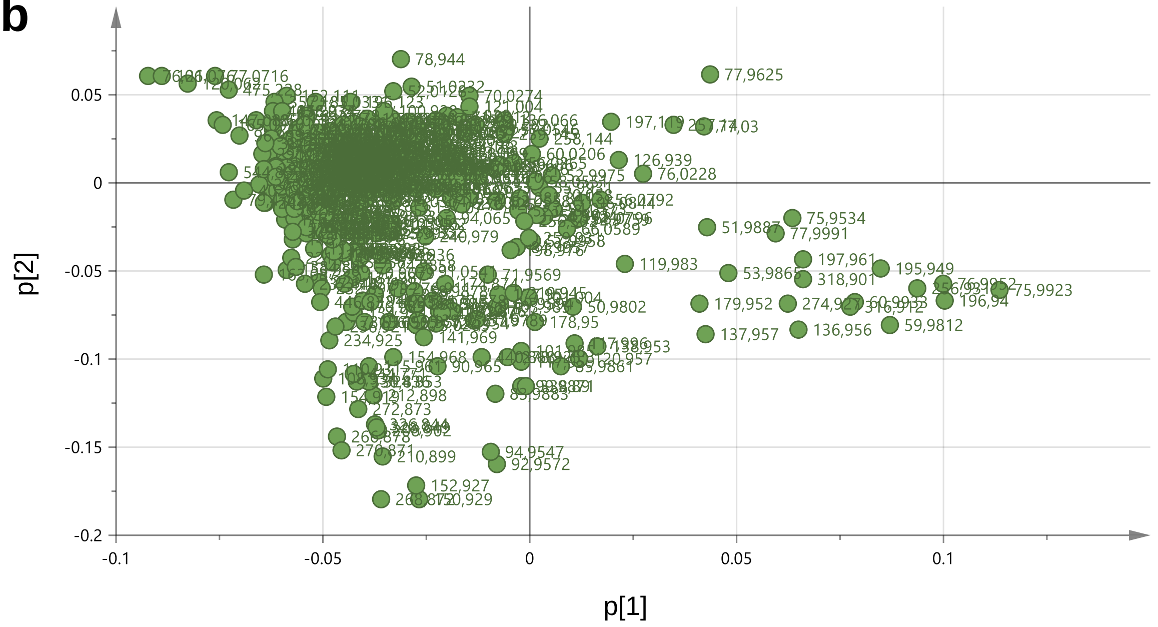

R2x[1] = 0.615 ; R2x[2] = 0.117

Figure S14: ToF-SIMS mass spectral data obtained from THP-1 cells exposed for 6 h to bare or silane-modified silica NPs analyzed using SIMCA software; (a) positive, and (b) negative ion mode.

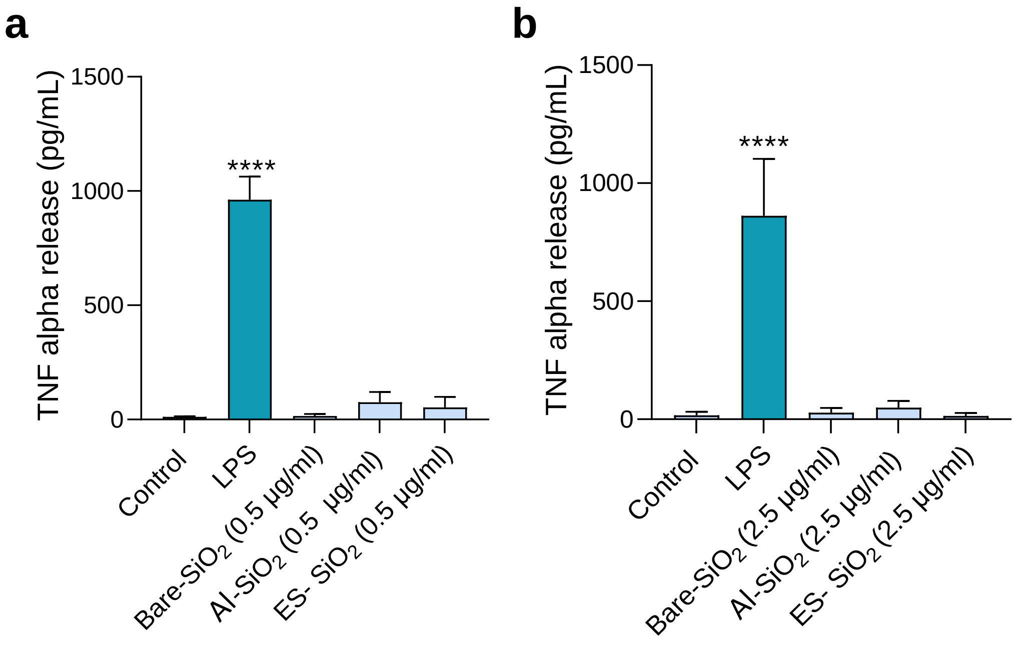

Figure S15: SiO_2 NPs do not trigger TNF-α production in monocytes. (a) Primary human CD14+ monocytes obtained from 3 individual donors were exposed for 6 h to SiO_2 as indicated (0.5 µg/mL). LPS (0.01 µg/mL) was included as a positive control. TNF-α production was determined by ELISA. (b) Monocyte-like THP-1 cells were exposed for 6 h to SiO_2 as indicated (2.5 µg/mL). LPS (0.01 µg/mL) was included as a positive control. TNF-α production was determined by ELISA. Data are mean values ± S.D. (n=3), ****$p < 0.0001$.

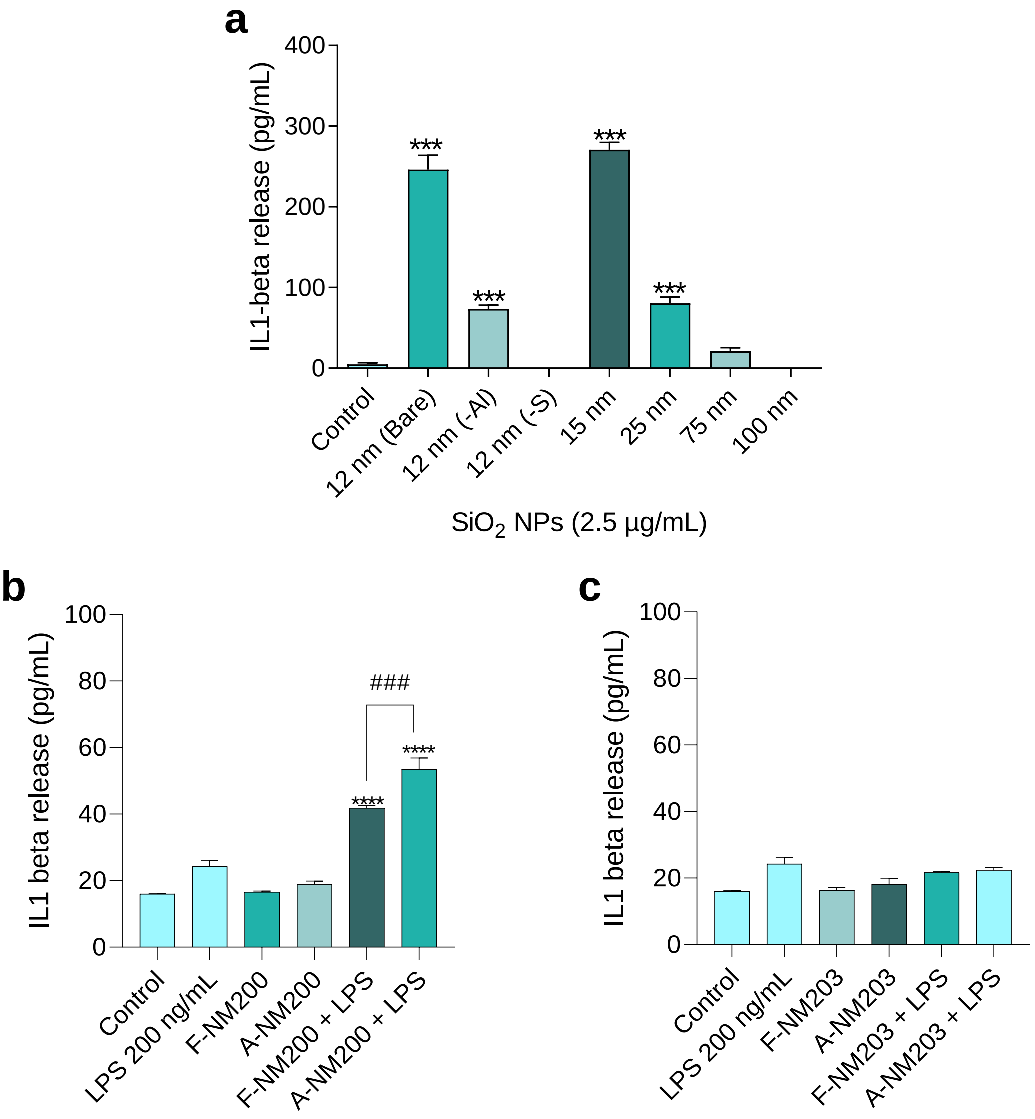

Figure S16: SiO_2 NPs trigger size- and surface-dependent IL-1β secretion. (a) IL-1β release in THP-1 cells after exposure to the indicated SiO_2 NPs for 6 h at 2.5 µg/mL. Note that the cells were not primed with LPS. (b) IL-1β release in THP-1 cells exposed to the benchmark SiO_2 NPs NM200 (50 µg/mL) with or without LPS priming. Note that the NPs were either freshly dispersed (denoted as "F") or had been aged for 18 months prior to exposure (denoted as "A"). (c) THP-1 cells exposed to the benchmark SiO_2 NPs NM203 (50 µg/mL) with or without LPS priming. The NPs were either fresh (F) or aged (A). No IL-1β secretion was observed. Data are shown as mean values ± S.D. (n=3). ***$p<0.001$, ****$p< 0.0001$, ###$p<0.001$.

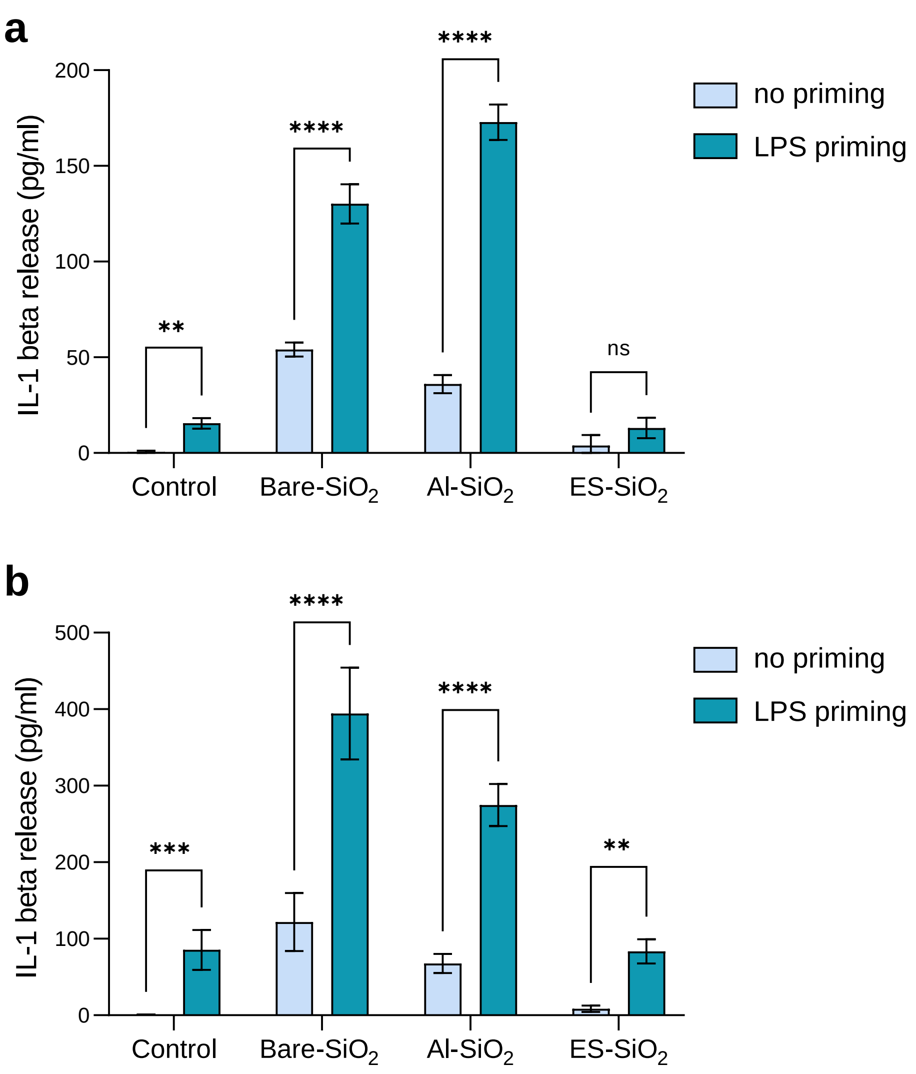

Figure S17: SiO_2 NP-triggered IL-1β production in primed *versus* non-primed conditions. (a) Non-differentiated (monocyte-like) THP-1 cells were exposed for 6 h to SiO_2 NPs as indicated (2.5 µg/mL) in the presence or absence of priming with LPS (0.1 µg/mL). (b) PMA-differentiated (macrophage-like) THP-1 cells were exposed for 6 h to SiO_2 NPs as indicated (2.5 µg/mL) in the presence or absence of priming with LPS (0.1 µg/mL). Data are shown as mean values ± S.D. (n=3). **$p<0.01$ ***$p<0.001$, ****$p<0.0001$.

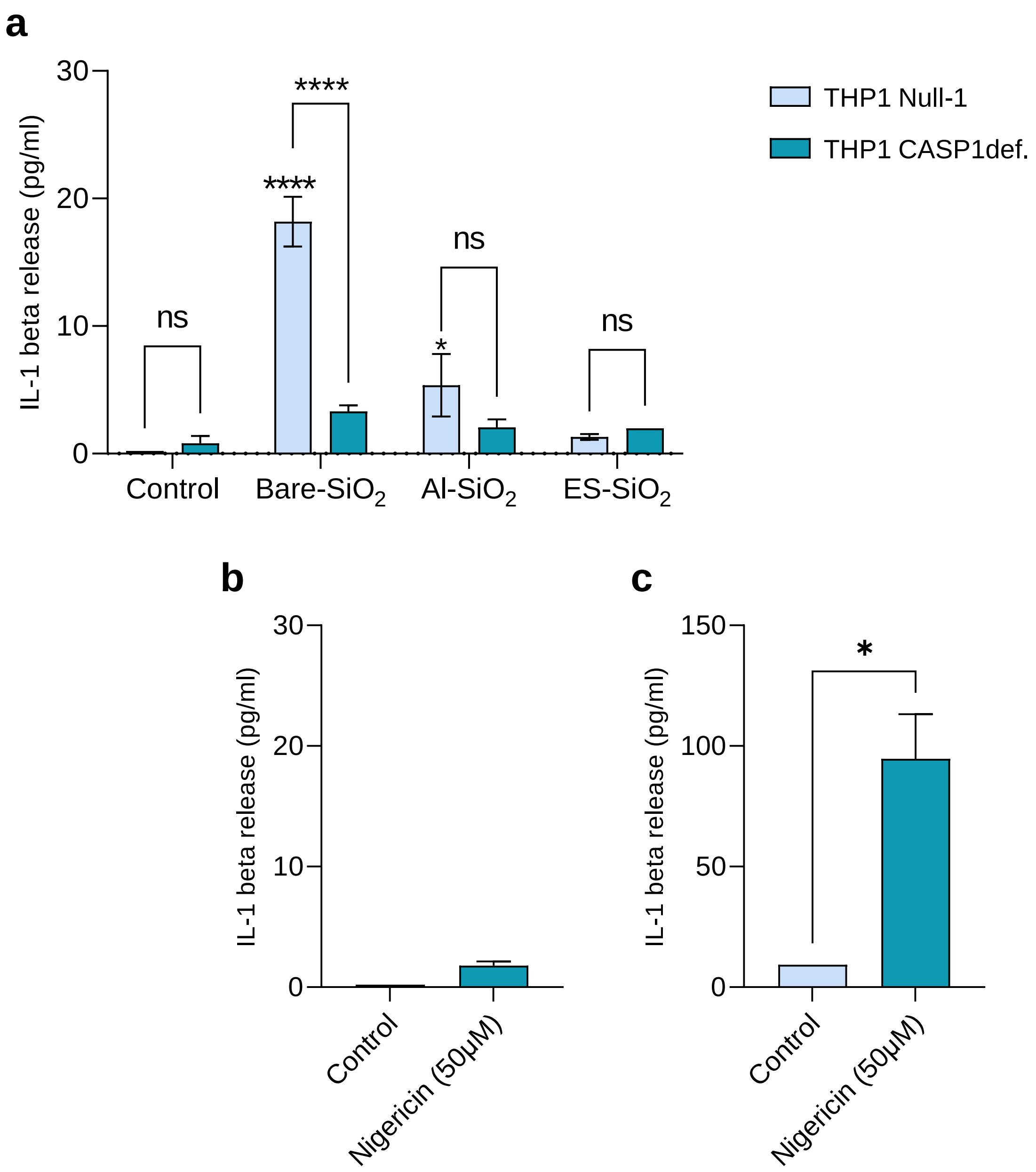

Figure S18: SiO_2 NP-triggered IL-1β production is caspase-1-dependent. (a) THP-1 control cells (Null-1) or caspase-1-deficient cells were exposed to bare *versus* Al-doped or silane modified silica NPs and IL-1β was determined by ELISA. The cells were not LPS primed. (b-c) The prototypic necroptosis inducer nigericin failed to elicit IL-1β release in non-primed cells (Null-1) (b) but IL-1β release was observed as expected following LPS priming (c). Data shown as mean values ± S.D. (n=3). *p<0.05, ****p< 0.0001.

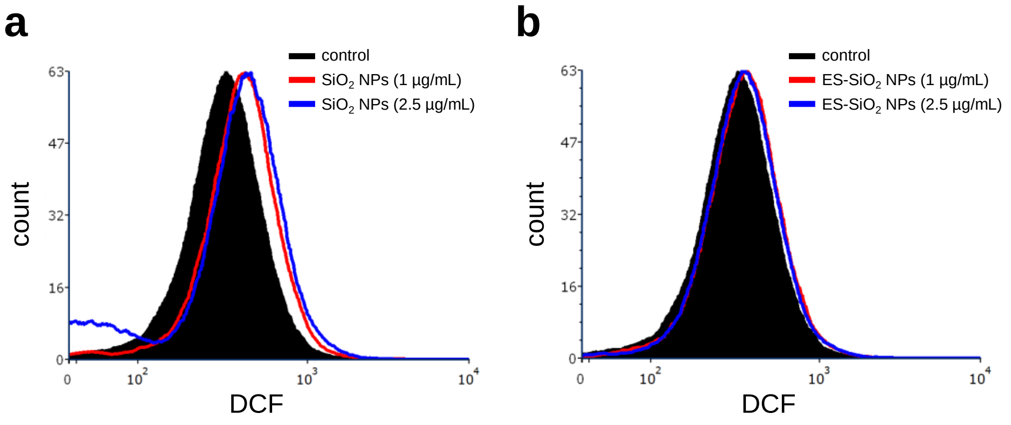

Figure S19: Uncoated silica NPs trigger cellular ROS production. THP-1 cells were exposed for 1 h to (a) uncoated or (b) silane-modified SiO_2 NPs (1 and 2.5 µg/mL) and ROS production was determined by flow cytometry using the DCF-DA assay. DCF-DA is a non-fluorescent compound that is internalized by cells and oxidized by cellular ROS into the fluorescent derivative 2’,7’-dichlorofluorescein (DCF).

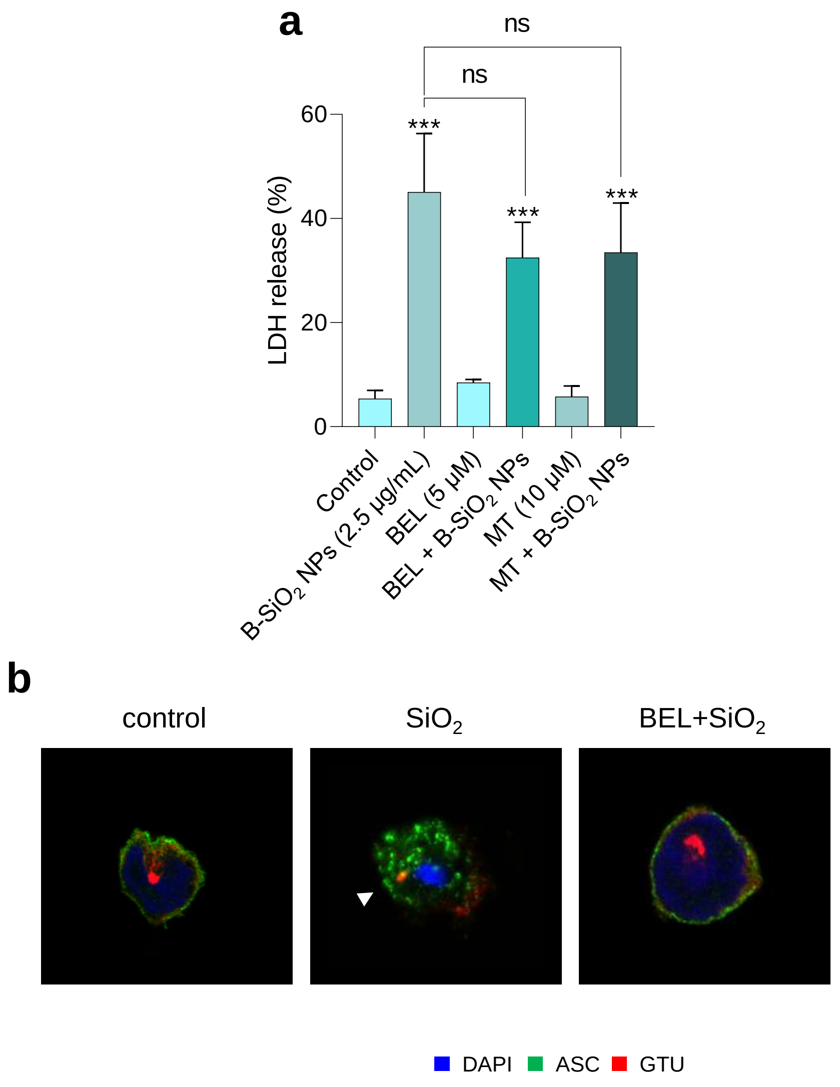

Figure S20: BEL prevents inflammasome assembly (and partially suppresses cell death). (a) THP-1 cells were exposed to uncoated or bare SiO_2 NPs (2.5 µg/mL) for 12 h in the presence or absence of BEL or MitoTEMPO (MT), a mitochondrially targeted scavenger of $O_2^{\bullet -}$. Cell death was determined using the LDH release assay. Data are shown are mean values ± S.D. (n=3). ***p<0.001. ns, not significant. (b) THP-1 cells were exposed for 6 h to the uncoated SiO_2 NPs in the presence or absence of BEL, a selective inhibitor of $iPLA_2$-VIA, and confocal imaging was performed to visualize ASC (a component of the NLRP3 inflammasome) and the centrosomal marker, γ-tubulin (GTU). Following SiO_2 NP exposure, ASC staining (green) was granular and co-localization with GTU (red) was detected (yellow) (arrow). However, BEL prevented the granular distribution of ASC and prevented the co-localization of ASC and GTU.
